# MicroRNA-204-5p is a tumor suppressor and potential therapeutic target in head and neck squamous cell carcinoma

**DOI:** 10.7150/thno.38507

**Published:** 2020-01-01

**Authors:** Zehang Zhuang, Pei Yu, Nan Xie, Yue Wu, Haichao Liu, Ming Zhang, Yifan Tao, Weiwang Wang, Hanqi Yin, Bin Zou, Jinsong Hou, Xiqiang Liu, Jiong Li, Hongzhang Huang, Cheng Wang

**Affiliations:** 1 Department of Oral and Maxillofacial Surgery, Guanghua School of Stomatology, Hospital of Stomatology, Sun Yat-sen University, Guangzhou 510055, China.; 2 Guangdong Provincial Key Laboratory of Stomatology, Sun Yat-sen University, Guangzhou 510080, China.; 3 Department of Oral Pathology, Guanghua School of Stomatology, Hospital of Stomatology, Sun Yat-sen University, Guangzhou 510055,China.; 4 Department of Operative Dentistry and Endodontics, Guanghua School of Stomatology, Hospital of Stomatology, Sun Yat-sen University, Guangzhou 510055, China.; 5 School of Life Science, Sun Yat-sen University, Guangzhou 510275, China.; 6 South China Institute of Biomedine, Guangzhou 510535, China.; 7 State Key Laboratory of Ophthalmology, Zhongshan Ophthalmic Center, Sun Yat-sen University, Guangzhou 510060, China.; 8 Department of Medicinal Chemistry, School of Pharmacy, and the Massey Cancer Center, Virginia Commonwealth University, Richmond, Virginia 23298-0540, United States.; 9 Institute for Structural Biology, Drug Discovery and Development, Virginia Commonwealth University, Richmond, Virginia 23298-0540, Unites States.

**Keywords:** miR-204-5p, tumor suppressor, metastasis, tumorigencity, head and neck squamous cell carcinoma

## Abstract

Elucidation of the molecular mechanisms governing aggressiveness of HNSCC may provide clinical therapeutic strategies for patients. In this study, a novel hub miR-204-5p functioning as tumor suppressor has been identified and explored in HNSCC.

**Methods**: A novel hub miR-204-5p was identified based on miRNA microarray, bioinformatics analysis and validated in different HNSCC patient cohorts. The functional role of miR-204-5p and its downstream and upstream regulatory machinery were investigated by gain-of-function and loss-of-function assays *in vitro* and *in vivo*. Interactions among miR-204-5p and SNAI2/SUZ12/HDAC1/STAT3 complex were examined by a series of molecular biology experiments. Then, the clinical relevance of miR-204-5p and its targets were evaluated in HNSCC samples. HNSCC patient-derived xenograft (PDX) model was used to assess the therapeutic value of miR-204-5p.

**Results**: We reveal that miR-204-5p as a tumor suppressor is commonly repressed in HNSCC, which can inhibit tumor growth, metastasis and stemness. Mechanically, miR-204-5p suppresses epithelial-mesenchymal transition (EMT) and STAT3 signaling by targeting SNAI2, SUZ12, HDAC1 and JAK2. Among these targets, we further showed that SNAI2, SUZ12, and HDAC1 form a repressive complex on CDH1 promoter to maintain EMT in HNSCC. In turn, the SNAI2/SUZ12/HDAC1 complex interacts with STAT3 on miR-204-5p-regulatory regions to suppress the transcription of miR-204-5p. Moreover, we also show that decrease of miR-204-5p indicates a poor prognosis in HNSCC patients and administration of agomiR-204-5p inhibits tumor growth and metastasis in HNSCC PDX models.

**Conclusion**: miR-204-5p-SNAI2/SUZ12/HDAC1/STAT3 regulatory circuit has a critical role in maintaining aggressiveness of HNSCC, suggesting that miR-204-5p might serve as a promising therapeutic target for clinical intervention.

## Introduction

Head and neck squamous cell carcinoma (HNSCC) is the most common epithelial malignancy of head and neck mucosa with high mobility and mortality. Currently, the standard treatment for HNSCC includes surgery with or without radiotherapy and chemotherapy 1-3. However, overall survival rate for HNSCC patients remains dismal due to rapid metastasis and high regional relapse rate 4, 5. Thus, it is of great urgency and significance to identify molecular mechanisms and effective therapies for HNSCC. MicroRNA (miRNA) belongs to a class of small noncoding RNAs containing 20 to 22 nucleotides that can regulate genes expression by binding to the 3' untranslated region (UTR) of target mRNAs at the posttranscriptional level 6, 7. Accumulating evidence suggests that miRNAs have been involved in cancer progression and may be seen as promising therapeutic targets 8-14. However, to our knowledge, limited studies have been performed to identify the cancer specific miRNAs and investigate their therapeutic potentials, especially with respect to HNSCC.

In this study, we have determined that miR-204-5p is a hub tumor suppressor miRNA that is markedly decreased and inversely correlated with lymph node metastasis (LNM) in HNSCC. Overexpression of miR-204-5p suppresses HNSCC tumor growth, metastasis and stemness through inhibiting epithelial-mesenchymal transition (EMT) and Janus kinase 2 (JAK2)/Signal transducer and activator of transcription 3 (STAT3) signaling by targeting SNAIL homolog 2 (SNAI2), suppressor of zeste 12 (SUZ12), histone deacetylase 1 (HDAC1) and JAK2 both *in vitro and in vivo*. Furthermore, we reveal that SNAI2, SUZ12, and HDAC1 form a repressive complex to maintain EMT in HNSCC. In turn, the SNAI2/SUZ12/HDAC1 complex interacts with STAT3 to suppress the transcription of miR-204-5p. Importantly, administration of agomiR-204-5p attenuated tumor growth and metastasis in HNSCC patient-derived xenograft models. Taken together, our results demonstrate that miR-204-5p functions as a tumor suppressor via the simultaneous repression of a cohort of pro-metastasis genes and may serve as a promising therapeutic target for HNSCC.

## Results

### MiR-204-5p is downregulated in HNSCC

**Figure 1:**
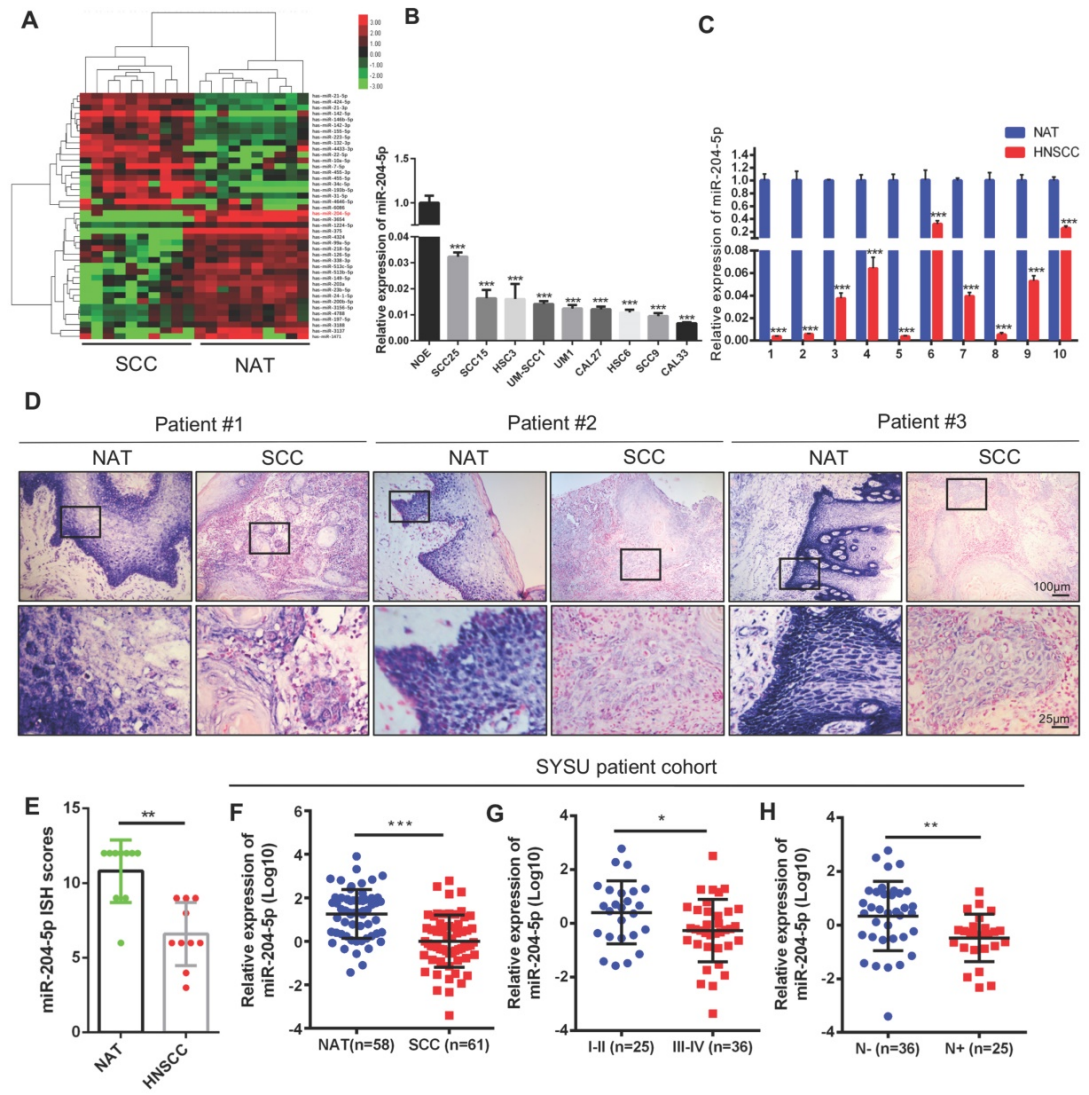
** Decrease of miR-204-5p correlates with malignant progression of HNSCC.** (A) Unsupervised hierarchical clustering of miRNAs which are differentially expressed in HNSCC and paired non-cancerous adjacent tissues (NAT) (fold changes > 2.0, *P* < 0.01). The red color scale (log2 fold change) represents a higher expression level, and the green color scale represents a lower expression level. (B) RT-qPCR analysis of miR-204-5p expression in normal oral epithelium and a panel of 9 HNSCC cell lines. (C) RT-qPCR analysis of miR-204-5p expression in 10 paired HNSCC samples and their corresponding NAT. (D) Representative images for miR-204-5p ISH staining in 10 paired HNSCC samples and their corresponding NAT. Three representative cases are shown. (E) miR-204-5p is downregulated in 10 HNSCC tumor specimens compared with paired NAT based on ISH. (F-H) RT-qPCR analysis of miR-204-5p expression in 58 NATs and 61 freshly collected human HNSCC samples (F), and the HNSCC samples were grouped by the clinical stage (G) and lymph node metastasis (H). Data represent mean ± SD. *P < 0.05, **P < 0.01 and ***P < 0.001 by Student's t test.

To screen miRNAs that are deregulated in HNSCC, we comparatively analyzed miRNA profiles by using 10 HNSCC patient samples and their paired non-cancerous adjacent counterparts. As shown in Figure 1A, microarray analysis revealed that 24 miRNAs were downregulated and 19 miRNAs were elevated in HNSCC samples when compared with non-cancerous adjacent tissues (NATs). Upregulated miR-21-5p, miR-21-3p, and downregulated miR-99a-5p have been shown to regulate malignant progression of HNSCC in our previous studies 15, 16. To further identify the hub miRNAs, described as the most closely associated with disease which have higher degree number compared with the other miRNAs in the global networks, Bayesian gene co-expression networks were constructed for the NATs (Figure S1A) and HNSCC group (Figure S1B) respectively. We found that two specific cancer associated hub miRNAs, miR-204-5p and miR-1224-5p, have the highest degree of interactions. Intriguingly, miR-204-5p was listed in the top 5 downregulated miRNAs (Figure S1C), indicating that deregulation of miR-204-5p might have a pivotal role in HNSCC progression. As expected, miR-204-5p was dramatically decreased in a panel of 9 HNSCC cell lines (Figure 1B) and in 10 HNSCC specimens (Figure 1C), respectively, as compared to the pooled normal human oral epithelium tissues and paired NATs. This result was further confirmed by in situ hybridization (ISH) analysis, showing that miR-204-5p staining was strongly detected in NATs and markedly reduced in tumor bulk (Figure 1D-E). Interestingly, the expression of miR-204-5p was significantly decreased in HNSCC tumor budding as compared to tumor bulk **(**Figure S1D-E), indicating a much more aggressive cancer subpopulation reported in our previous studies 17-19. To further validate these findings, 58 NATs and 61 HNSCC tissue samples were employed to detect the expression of miR-204-5p by RT-qPCR. The results shown in Figure 1F confirm that expression of miR-204-5p was dramatically decreased in HNSCC as compared to NATs. More importantly, miR-204-5p was inversely associated with clinical stage and LNM in HNSCC (Figure 1G-H). Strikingly, we noticed that the expression of miR-204-5p was decreased in 52 patients among the 58 HNSCC samples as compared to their paired NATs (Figure S1F). Similar results were also observed in the TCGA patient cohort (Figure S1G-I). Collectively, our data confirm that miR-204-5p is markedly downregulated and associated with malignant progression in HNSCC.

### MiR-204-5p inhibits cell proliferation, migration, invasion and stem cell-like properties of HNSCC *in vitro*

**Figure 2:**
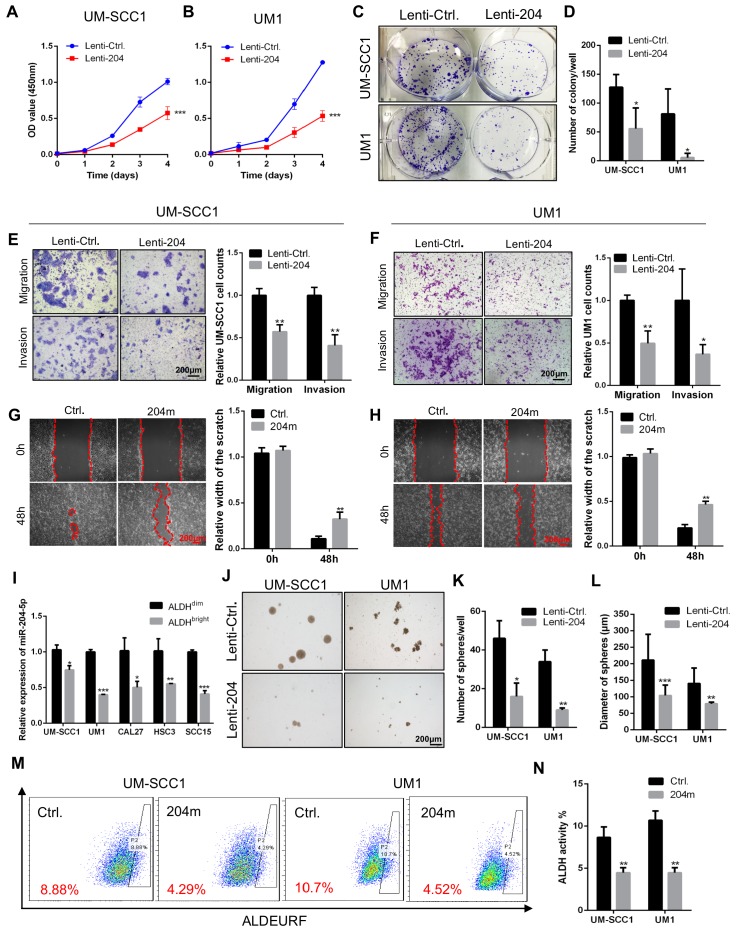
** MiR-204-5p inhibits cell proliferation, migration, invasion and stem cell-like properties of HNSCC *in vitro.*** (A, B) Cell proliferation was measured by CCK8 assay in HNSCC cells transfected with Lenti-miR-204-5p. Data represent mean ± SD. ***P < 0.001 by two-way ANOVA test. (C, D) Overexpression of miR-204-5p suppressed tumor growth of HNSCC cells by colony formation assay. (E, F) Overexpression of miR-204-5p inhibited cell migration and invasion in HNSCC cells. (G, H) The ability of cell motility was evaluated by wound healing assay in HNSCC cells treated with miR-204-5p mimics and control mimics. (I) RT-qPCR showed that miR-204-5p expression was decreased in ALDHbright cancer stem cells of HNSCC as compared to ALDHdim non-cancer stem cells. (J-L) Spheroid formation assay showed that the self-renewal ability was decreased in miR-204-5p overexpressing cells. (M, N) ALDEURF assay showed that ALDHbright cancer stem cell population was reduced in HNSCC cells treated with miR-204-5p mimics. Data represent mean ± SD. *P < 0.05, **P < 0.01 and ***P < 0.001 by Student's t test.

To address the role of miR-204-5p in HNSCC cells, gain or loss-of-functional analyses were performed by using miR-204-5p mimics (204m) and inhibitors (204i) or lenti-virus mediated stable overexpression of miR-204-5p (Lenti-204) in human HNSCC cell lines UM-SCC1 and UM1. The miR-204-5p transfection efficiency was determined by RT-qPCR (Figure S2A-B). As shown in Figure 2A-D, overexpression of miR-204-5p led to a decrease of cell proliferation and clonogenic activities. Moreover, cell migratory and invasive capacities were dramatically reduced in cells overexpressing miR-204-5p (Figure 2E-F) when comparing with control cells. Similarly, cell motility was also repressed in HNSCC cells transfected with miR-204-5p mimics (Figure 2G-H). Conversely, miR-204-5p depletion significantly promoted cell migration and invasion relative to the negative control (Figure S2C-D). To further investigate whether miR-204-5p is involved in stem cell-like properties of HNSCC, RT-qPCR analysis was performed to detect the expression of miR-204-5p in FACSorted cancer stem cells. This analysis showed that miR-204-5p was significantly decreased in ALDH^bright^ cancer stem cells as compared to ALDH^dim^ non-cancer stem cells of HNSCC (Figure 2I). Functionally, the spheroid formation assay showed that self-renewal ability was decreased in cells overexpressing miR-204-5p (Figure 2J-L) and a reduction of ALDH1^bright^ cells population was observed in cells that were treated with miR-204-5p mimics (Figure 2M-N), indicating that the capacity for initiating cancer was impaired. These results indicated that miR-204-5p functions as a tumor suppressor in HNSCC.

### MiR-204-5p inhibits tumor growth, metastasis and tumorigencity of HNSCC *in vivo*

**Figure 3:**
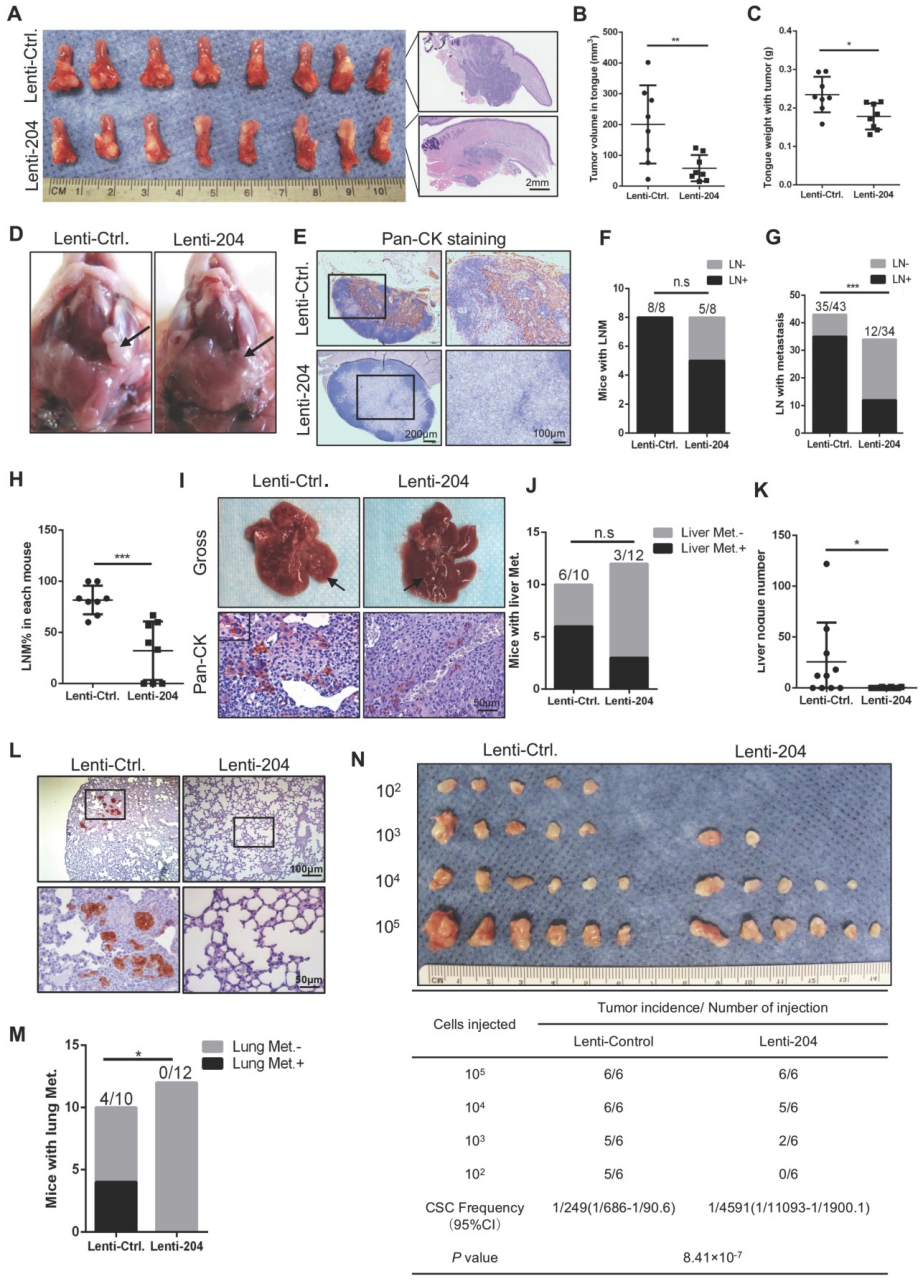
** MiR-204-5p inhibits tumor growth, metastasis and tumorigenesis of HNSCC *in vivo.*** (A) Representative image of HNSCC orthotopic xenograft inoculated with the indicated cells and histopathological analysis of the tumor (n=8 per group). Tumor volume (B) and weight (C) were inhibited in mice bearing miR-204-5p overexpressing cells as compared to mice bearing control cells. *P < 0.05 and **P < 0.01 by Student's t test. (D) Representative image of cervical lymph node from mice bearing miR-204-5p overexpressing cancer cells and their corresponding control cells. (E) Representative image of cervical lymph node examined by Pan-CK staining. (F) The percentage of mice having lymph node metastasis was analyzed by Fisher's exact test. n.s indicates non-significant. (G) The percentage of lymph node with metastatic tumor cells was analyzed by Fisher's exact test. ***P < 0.001. (H) The percentage of lymph node with metastatic tumor cells in each mouse. ***P < 0.001 by Student's t test. (I) Representative image of liver metastasis in nude mice inoculated with the indicated cells. Pan-CK staining was used to detect the metastatic tumor cells. (J) The percentage of mice with liver metastasis was analyzed by Fisher's exact test. n.s indicates non-significant. (K) The liver nodule number was analyzed in nude mice inoculated with miR-204-5p overexpressing cells and control cells. n=10-12 *P < 0.05 by Student's t test. (L) Representative image of lung metastasis examined by pan-CK staining. (M) The percentage of mice with lung metastasis was analyzed by Fisher's exact test. n=10-12. *P < 0.05. (N) *In vivo* limiting dilution analysis of HNSCC cells transfected with miR-204-5p. The frequency of allograft formation at each cell dose injected is shown.

To validate the suppressive role of miR-204-5p in HNSCC *in vivo*, the HNSCC orthotopic xenograft model was established with the miR-204-5p overexpressing cancer cells and their corresponding control cells, which were injected into the tongues of nude mice. As shown in Figure 3A-C, mice bearing cells overexpressing miR-204-5p displayed smaller tumors with lower tumor volume and weight than the mice bearing control cells. Moreover, the lymph node metastasis was significantly decreased in mice bearing cells overexpressing miR-204-5p when compared with mice bearing control cells (Figure 3D-H). Next, the distant metastasis model was established by injecting cells through the tail veins of nude mice. Six of ten mice (60%) bearing control cells developed liver metastasis, while only three of twelve mice (25%) bearing cancer cells overexpressing miR-204-5p did (Figure 3I-J). Importantly, the number of liver metastatic tumor nodules was dramatically decreased in mice with the miR-204-5p overexpression as compared to control mice (Figure 3K). Furthermore, the incidence of lung metastases was also reduced in these mice compared with control mice (Figure 3L-M).

To further evaluate the effect of miR-204-5p on tumorigenicity of HNSCC cells, a limiting-dilution assay was performed and four doses (10^5^, 10^4^, 10^3^ and 10^2^) of cells overexpressing miR-204-5p and their corresponding control cells were subcutaneously inoculated in BALB/c nude mice. As shown in Figure 3N, miR-204-5p-transduced cancer cells displayed lower tumorigenicity and cancer stem cell frequency than did in control cells. Of particular note, the miR-204-5p overexpressing cancer cells could not form visible tumors when 10^2^ cells were injected, suggesting that miR-204-5p repressed the tumor initiating cell population in HNSCC cells. These results were consistent with our findings *in vitro*, and supported the notion that miR-204-5p strongly inhibits the tumorigenesis, growth and metastasis of HNSCC.

### MiR-204-5p suppresses EMT and STAT3 pathway

**Figure 4:**
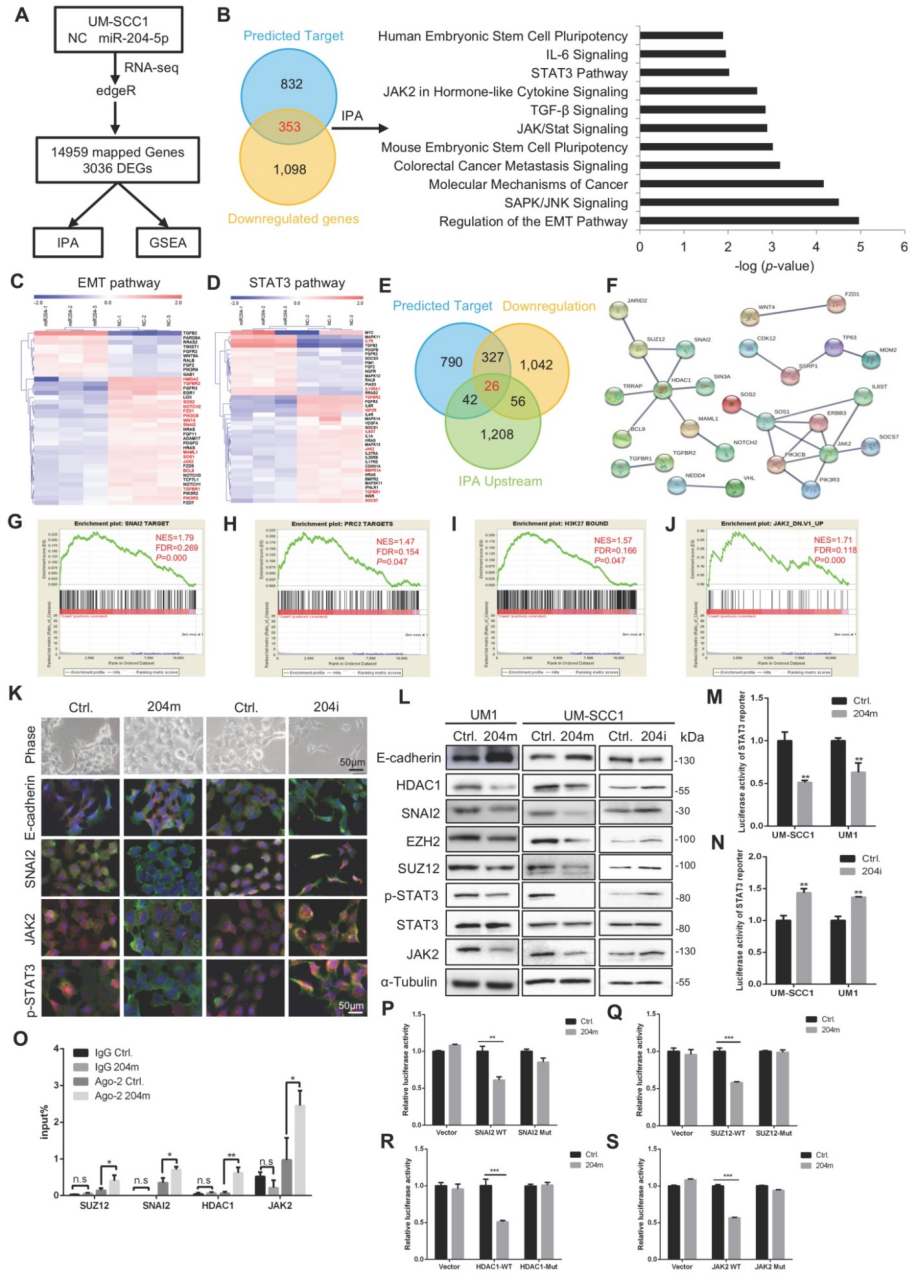
** MiR-204-5p suppresses EMT and STAT3 pathway by targeting SNAI2, SUZ12, HDAC1 and JAK2.** (A) Experimental scheme of RNA-Seq in UM-SCC1 cells and subsequent data analysis. (B) IPA showing that miR-204-5p target genes were enriched in metastasis and stemness associated pathway, such as EMT and STAT3 pathway. Heat map of representative EMT (C) or STAT3 pathway (D) related molecules from UM-SCC1 RNA-Seq results. Red font indicates the predicted miR-204-5p targets. (E) Venn diagram showing the predicted miR-204-5p target genes served as upstream regulator based on RNA-seq, IPA and StarBase prediction. (F) PPI prediction among potential targets by using STRING database. GSEA reveals positive enrichment of miR-204-5p-regualted genes in SNAI2 target gene sets (G), PRC2 target gene sets (H), H3K27 bound gene sets (I) and genes up-regulated after knockdown of JAK2 (J). (K) Morphological and Immunofluorescence analysis of cells treated with miR-204-5p mimics and inhibitors. The expression of E-cadherin, SNAI2, JAK2, p-STAT3 (Red) and β-actin (Green) were detected. (L) WB of E-cadherin, HDAC1, SNAI2, EZH2, SUZ12, p-STAT3, STAT3 and JAK2 in HNSCC cells treated with miR-204-5p mimics or inhibitors. STAT3 reporter activities were measured in HNSCC cells treated with miR-204-5p mimics (M) and inhibitors (N). (O) RIP analysis revealed that SUZ12, SNAI2, HDAC1 and JAK2 mRNA were recruited to miRNP complex. Luciferase activity of reporters with wild type or mutant 3'UTRs of SNAI2 (P), SUZ12 (Q), HDAC1 (R) and JAK2 (S) in HNSCC cells co-transfected with indicated oligonucleotides. Data represent mean ± SD. *P < 0.05, **P < 0.01*, P <* 0.001 by Student's t test.

To understand the molecular mechanisms underlying miR-204-5p mediated suppression of HNSCC progression, we performed RNA-seq analysis in UM-SCC1 cells transfected with miR-204-5p mimics and control mimics. Of the 14959 mapped genes, 3036 differentially expressed genes (DEGs) were identified (Figure 4A), including 1451 downregulated and 1585 up-regulated genes (Supplementary data 1). Ingenuity Pathway Analysis (IPA) in the 3036 DEGs revealed that signal transduction and activation of the transcription 3 (STAT3) pathway, the molecular mechanism of cancer, JAK/STAT signaling, IL6 signaling and mouse embryonic stem cell pluripotency were all significantly affected by miR-204-5p (Figure S3A**),** implicating that miR-204-5p in a critical role of maintaining cancer stem cell-like traits, which was confirmed in our earlier functional studies above. On the other hand, the miR-204-5p associated DEGs were involved in Rac signaling, colorectal cancer metastasis signaling and TGFβ signaling (Figure S3A), a well-known regulator of epithelia-mesenchymal transition (EMT), supporting the observations that miR-204-5p inhibited invasion and metastasis. Moreover, several growth factor signaling pathways including IGF, EGF, NGF, PDGF, HGF, ERK5, SAPK/JNK, PI3K/AKT (Figure S3B), were significantly regulated by miR-204-5p, indicating that miR-204-5p inhibits cell cycle progression. These results supported our earlier observations that miR-204-5p suppresses cell proliferation and tumor growth. To further investigate the potential pathway directly regulated by miR-204-5p target genes, IPA was performed again by using the overlapped genes from the predicted miR-204-5p target genes and the down-regulated genes upon miR-204-5p overexpression. We found that metastasis associated pathway and stemness related pathway were significantly enriched in miR-204-5p target genes, such as EMT pathway, colorectal cancer metastasis signaling, TGFβ signaling, mouse embryonic stem cell pluripotency, human embryonic stem cell pluripotency, STAT3 pathway and IL-6 signaling (Figure 4B), which was consistent with the IPA analysis by using the whole DEGs. Collectively, these analyses indicated that miR-204-5p target genes dominantly regulate EMT and the properties of cancer stem cells. Interestingly, among the miR-204-5p target genes, the expression of JAK2, TGFBR1 and TGFBR2, involved in both EMT and STAT3 signaling, were all decreased in cells treated with miR-204-5p mimics (Figure 4C-D), suggesting that these genes might drive the interaction of EMT and stemness signaling. Unexpectedly, the epithelial markers displayed minimal alterations in our RNA-seq data, suggesting that miR-204-5p mainly suppressed the expression of mesenchymal genes and had a crucial role in regulating partial EMT, which has been confirmed by single cell RNA-seq to play a major role in HNSCC metastasis 20.

Next, to identify the novel targets of miR-204-5p which can be served as the upstream regulators involved in EMT and STAT3 pathway and investigate their potential interactions, we performed the IPA upstream regulator analyses. Then, the IPA upstream regulators and predicted targets were merged with miR-204-5p-repressed genes, yielding a total of 26 candidate upstream targets of miR-204-5p (Figure 4E). Finally, the STRING analysis was done by using EMT and STAT3 pathway related targets and 26 candidate upstream targets to investigate their potential interactions. As shown in Figure 4F, two major clusters characterized with HDAC1 or JAK2 were established. Notably, among these candidates, SNAI2, a well-known EMT transcriptional factor and SUZ12, a subunit of polycomb repressive complex, have been identified to regulate EMT and be upregulated in HNSCC 20-23. HDAC1, the major histone diacetylase, has been proven to cooperate with SNAI1/SNAI2 and EZH2 to regulate EMT 24, 25. Further, this analysis also strongly suggested that SNAI2, SUZ12 and HDAC1 might form a repressor complex, implicating the interaction of transcriptional factors and histone modification regulators to have a pivotal role in miR-204-5p-mediated inhibition of cancer progression. In addition, as predicted, JAK2 was a key direct target of miR-204-5p and mediated the classical JAK2/STAT3 signaling. In support, GSEA demonstrated that the gene expression changes induced by miR-204-5p were positively correlated with signatures of the SNAI2 targets (Figure 4G), PRC2 targets (Figure 4H), H3K27 bounded genes (Figure 4I) and the genes up-regulated after knockdown of JAK2 (Figure 4J). These findings indicated that miR-204-5p might directly target a SUZ12/SNAI2/HDAC1 complex and JAK2/STAT3 signaling to suppress HNSCC progression.

### MiR-204-5p targets SNAI2, SUZ12, HDAC1 and JAK2

To further confirm that miR-204-5p regulated EMT and STAT3 signaling, immunofluorescence staining was performed in UM-SCC1 cells treated with miR-204-5p mimics or inhibitors. As shown in Figure 4K, the expression of SNAI2, JAK2 and p-STAT3 were all decreased in cells treated with miR-204-5p mimics (204m), but increased in cells treated with miR-204-5p inhibitors (204i). The expression of E-cadherin was significantly increased and translocated to cell membrane in cells transfected with miR-204-5p mimics, suggesting that the epithelial marker could be partially restored by overexpressing miR-204-5p. Similar results were observed in qPCR and western blotting analysis, and all the predicted miR-204-5p targets were significantly inhibited in cells treated with miR-204-5p mimics but increased in cells treated with miR-204-5p inhibitors, including SNAI2, SUZ12, HDAC1 and JAK2 (Figure 4L and Figure S3C-D). Strikingly, the expression of EZH2 was found to be suppressed in cells treated with miR-204-5p mimics and increased in cells transfected with inhibitors, indicating that the PRC2 activity was strongly regulated by miR-204-5p. Consistently, the luciferase reporter assay confirmed that the activity of STAT3 was significantly suppressed in cells treated with miR-204-5p mimics and enhanced in cells treated with miR-204-5p inhibitors (Figure 4M-N).

To validate the miR-204-5p-directed RISC binding to SUZ12, SNAI2, HDAC1 and JAK2 mRNA, the RIP-PCR assay was performed. SUZ12, SNAI2, HDAC1 and JAK2 mRNA fragments were significantly enriched in the Ago2 co-immunoprecipitation (co-IP) fraction in cells treated with miR-204-5p mimics (Figure 4O**)**. Next, dual-luciferase reporter assays were performed using constructs containing these targeting sites (Figure S3E). As illustrated in Figure 4P-S, the luciferase activities of the construct containing the miR-204-5p targeting sites from the 3'-UTR of SUZ12, SNAI2, SUZ12, HDAC1 and JAK2 were significantly reduced in cells transfected with miR-204-5p mimics, and site-specific mutagenesis of the miR-204-5p binding sites abrogated the miR-204-5p-mediated suppression of the reporter activities. These findings confirm that miR-204-5p suppresses EMT and JAK2/STAT3 signaling and directly targets SUZ12, SNAI2, HDAC1 and JAK2 mRNA.

### MiR-204-5p inhibits the recruitment of SNAI2/PRC2/HDAC1 complex to CDH1 promoter

**Figure 5:**
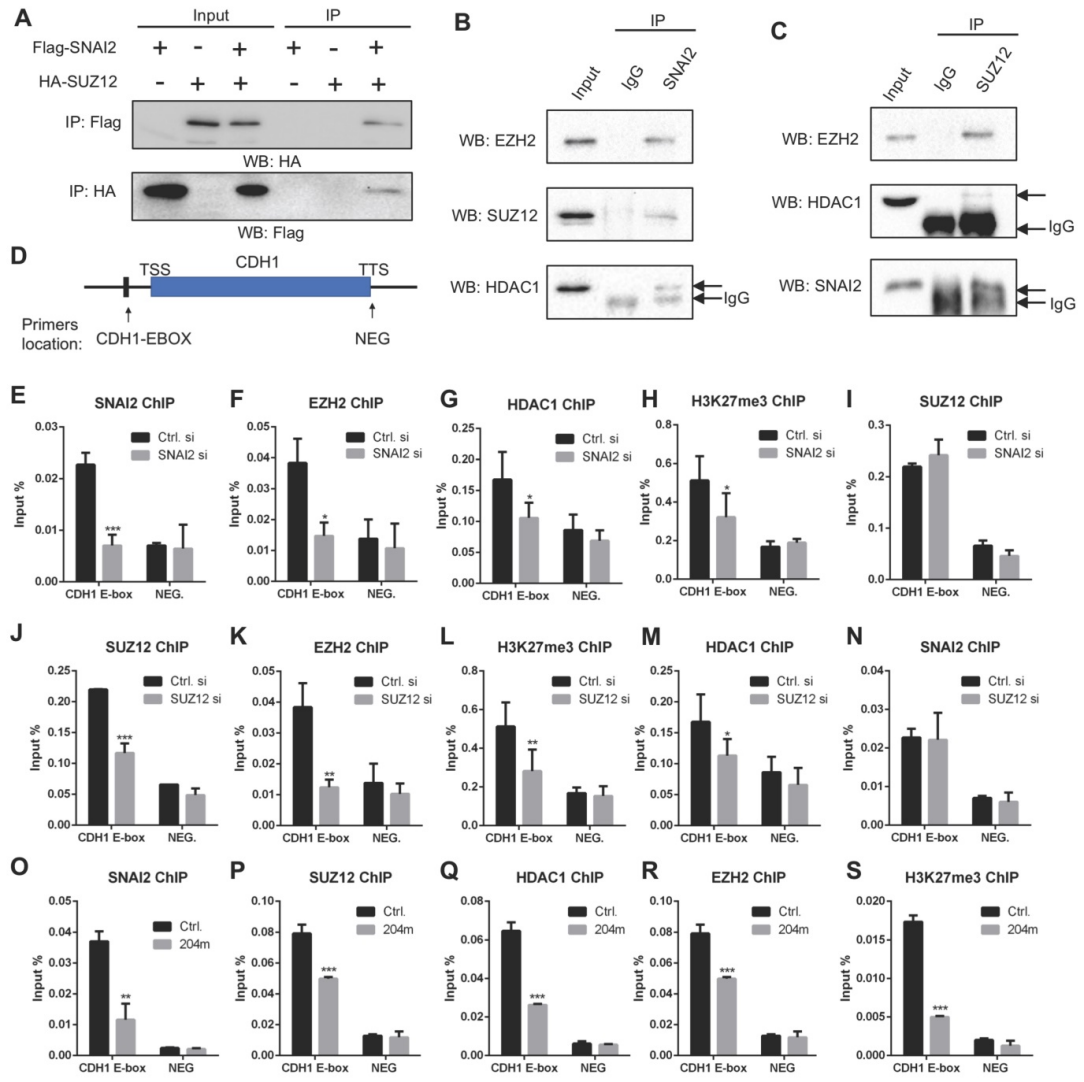
** MiR-204-5p inhibits the recruitment of SNAI2/PRC2/HDAC1 complex to CDH1 promoter.** (A) IP analysis showed that exogenous SUZ12 and SNAI2 formed a complex. (B) IP with SNAI2 showed that endogenous SNAI2 physically interacted with endogenous EZH2, SUZ12 and HDAC1. (C) IP with SUZ12 demonstrated that endogenous SUZ12 associated with endogenous EZH2, HDAC1 and SNAI2. (D) A schematic view of ChIP primer location in CDH1 gene. Enrichments of SNAI2 (E), EZH2 (F), HDAC1 (G), H3K27Me3 (H) and SUZ12 (I) to CDH1 promoter in cells treated with SNAI2 siRNAs. Enrichments of SUZ12 (J), EZH2 (K), H3K27Me3 (L), HDAC1 (M) and SANI2 (N) to CDH1 promoter in cells treated with SUZ12 siRNAs. Enrichments of SNAI2 (O), SUZ12 (P), HDAC1 (Q), EZH2 (R) and H3K27Me3 (S) to CDH1 promoter in cells treated with miR-204-5p mimics. Data represent mean ± SD. *P < 0.05, **P < 0.01, ***P < 0.001 by Student's t test.

Next, we asked how these miR-204-5p targets regulated the downstream EMT signaling. Previously, our STRING analysis suggested that SNAI2/SUZ12/HDAC1 might form a complex to regulate expression of target genes. Moreover, the transcriptional repressor SNAI1 and its close related gene SNAI2 are essential regulators for EMT. It has been reported that SNAI1 could interact with transcriptional repressor such as the PRC2 complex and HDAC1 on the EMT target gene promoter to suppress gene transcription 24. Such transcriptional repression plays a key role in maintaining the EMT phenotype. Our data confirmed that SUZ12, HDAC1 and SNAI2 are the direct targets of miR-204-5p. Therefore, we hypothesized that the SNAI2/HDAC1/SUZ12 complex was necessary for E-cadherin repression to maintain EMT signaling and miR-204-5p inhibited EMT by targeting this complex in HNSCC. To verify this hypothesis, we first examined whether SNAI2 could interact with HDAC1 and PRC2 complex. Co-IP assays in HEK293T cells showed the interaction between Flag-tagged SNAI2 and HA-tagged SUZ12 (Figure 5A). Then, the interactions among endogenous SNAI2, HDAC1, SUZ12, EZH2 were verified in UM-SCC1 cells (Figure 5B-C). These results supported the notion that PRC2, SNAI2 and HDAC1 could form such a co-repressor complex.

Then, we performed ChIP-qPCR experiments for SNAI2, SUZ12, HDAC1, EZH2 and H3K27me3 using crosslinked chromatin from UM-SCC1 cells treated with or without SNAI2 siRNAs. The enriched DNA from the immunoprecipitates (IPs) was quantified by RT-PCR using primers spanning the E-Box regions of the CDH1 promoter (Figure 5D), which is a well-characterized EMT hallmark. As a strict negative control, a region located in the transcriptional terminal site of CDH1 gene (NEG) was also examined. As shown in Figure 5E-H, enrichments of SNAI2, HDAC1, EZH2 and H3K27me3 were significantly decreased in UM-SCC1 cells treated with SNAI2 siRNAs. Of note, SUZ12 enrichment was not significantly changed in SNAI2 siRNAs treated cells, indicating that SUZ12 recruitment was independent of SNAI2 (Figure 5I). To clarify the role of SUZ12 in the CDH1 promoter, we silenced the endogenous SUZ12 and found that the enrichments of EZH2, HDAC1 and H3K27me3 were significantly decreased but not the SNAI2, suggesting that the SUZ12 was crucial for EZH2 and HDAC1 recruitment to CDH1 promoter (Figure 5J-N). Importantly, the enrichments of SNAI2, SUZ12, EZH2, HDAC1 and H3K27me3 were all significantly decreased in cells treated with miR-204-5p mimics (Figure 5O-S), confirming that miR-204-5p inhibits the enrichment of SNAI2/PRC2/HDAC1 complex to CDH1 promoter. In turn, SNAI2/PRC2/HDAC1 complex suppressed the expression of CDH1 at transcriptional level. Altogether, these data showed that miR-204-5p restores E-cadherin expression through targeting the SNAI2/PRC2/HDAC1 repressor complex.

### MiR-204-5p is suppressed by SNAI2/ PRC2/HDAC1/STAT3 complex

**Figure 6:**
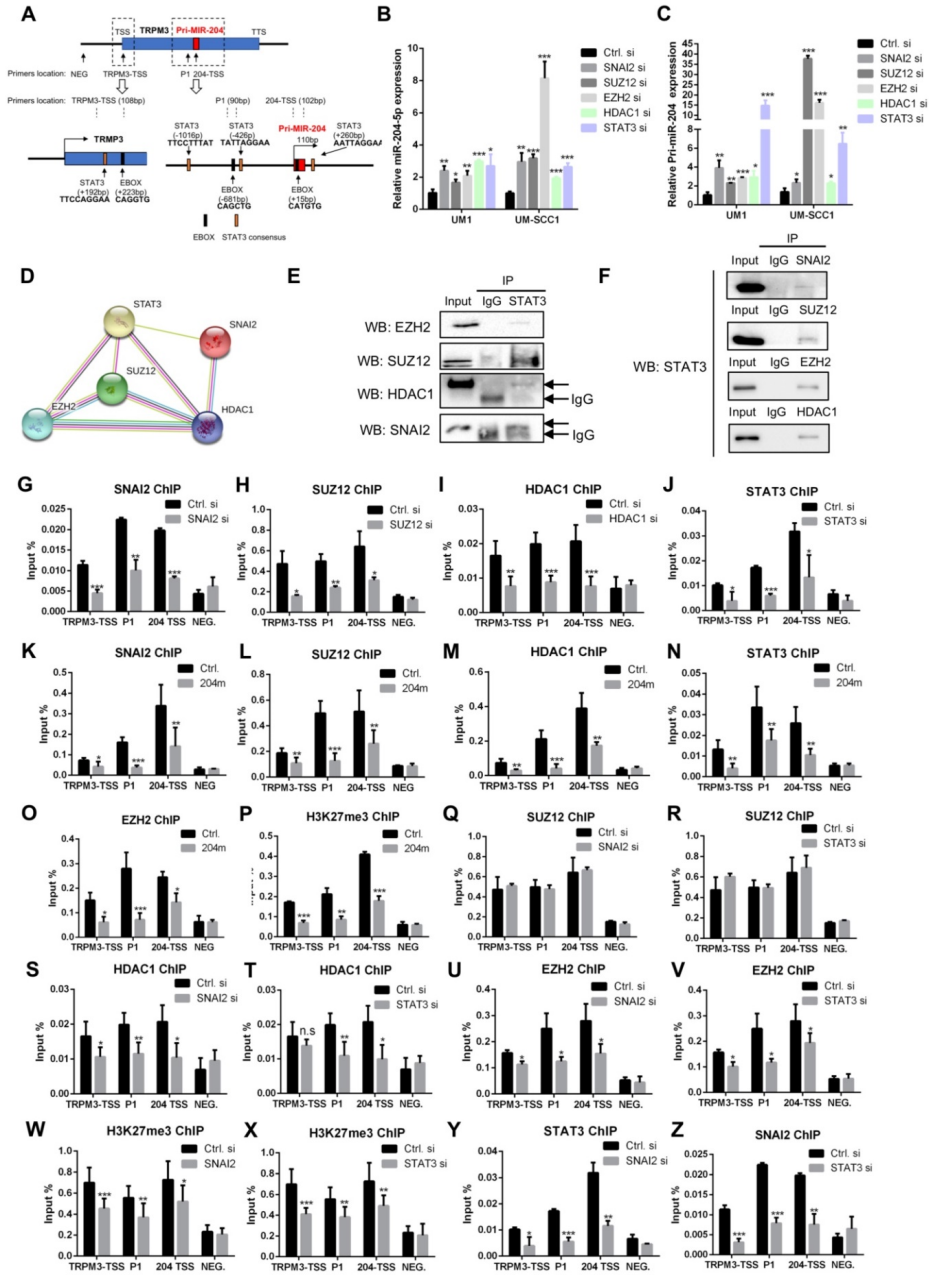
** MiR-204-5p is suppressed by SNAI2/PRC2/HDAC1 complex and STAT3.** (A) Scheme of E-box, STAT3 binding site and ChIP primer location in TRPM3 gene. RT-qPCR analysis of miR-204-5p (B) or pri-miR-204 (C) expression in HNSCC cells treated with SNAI2, SUZ12, EZH2, HDAC1 and STAT3 siRNAs. (D) PPI prediction showed that SNAI2, SUZ12, EZH2, HDAC1 and STAT3 might form a complex by using STRING database. (E, F) IP analysis demonstrated that STAT3 physically interacted with SNAI2, SUZ12, HDAC1 and EZH2. ChIP analysis showed that SNAI2 (G), SUZ12 (H), HDAC1 (I) and STAT3 (J) were enriched in the promoter of TRPM3 and pri-miR-204 genes. Enrichments of SNAI2 (K), SUZ12 (L), HDAC1 (M), STAT3 (N), EZH2 (O) and H3K27me3 (P) to the promoter of TRPM3 and pri-miR-204 in cells treated with miR-204-5p mimics. Enrichments of SUZ12 to the indicated locations in cells treated with SNAI2 (Q) and STAT3 (R) siRNAs. Enrichments of HDAC1 to the indicated locations in cells treated with SNAI2 (S) and STAT3 (T) siRNAs. Enrichments of EZH2 to the indicated locations in cells treated with SNAI2 (U) and STAT3 (V) siRNAs. Enrichments of H3K27me3 to the indicated locations in cells treated with SNAI2 (W) and STAT3 (X) siRNAs. Enrichments of STAT3 (Y) and SNAI2 (Z) to the indicated locations in cells treated with SNAI2 and STAT3 siRNAs, respectively. Data represent mean ± SD. *P < 0.05, **P < 0.01 and ***P < 0.001 by Student's t test. n.s indicates non-significant.

Previously, studies revealed that miRNAs were epigenetically regulated in several cancers 26-28. Interestingly, E-box and STAT3 biding sites were found in the promoter of pri-miR-204 (Figure 6A), suggesting that the SNAI2/PRC2/HDAC1 repressor complex and STAT3 might regulate miR-204-5p expression. Indeed, our qPCR results showed that the expression of miR-204-5p and pri-miR-204 is significantly increased in cells treated with SNAI2, SUZ12, EZH2, HDAC1 and STAT3 siRNAs as compared to the control siRNA (Figure 6B-C). The restoration of miR-204-5p was also observed in HNSCC cells treated with HDAC1 inhibitors (SAHA) and EZH2 inhibitors (GSK126) (Figure S4A-B). Similar results were observed for TRPM3 expression, the host gene of miR-204-5p (Figure S4C-D). Interestingly, the suppression of miR-204-5p mediated by STAT3 at transcriptional level was observed. As shown in Figure 6A, STAT3 binding sites occurred in the vicinity of a canonical E-box binding motif (CATGTG-15bp downstream from the pri-miR-204 transcriptional start site) and a non-canonical E-box binding site (CAGCTG-681bp upstream form the pri-mir-204 transcriptional start site), indicating that E-box mediated recruitment SNAI2/PRC2/HDAC1 complex is involved in STAT3-mediated miR-204-5p repression. Then, STRING analysis showed that STAT3 interacted with SNAI2, SUZ12, EZH2 and HDAC1 (Figure 6D), which was further confirmed by a co-IP assay (Figure 6E-F).

Next, to determine whether the miR-204-5p promoter recruited the SNAI2/PRC2/HDAC1 repressor complex and STAT3, we performed ChIP experiments for SNAI2, SUZ12, HDAC1, and STAT3 using crosslinked chromatin from HNSCC cells. The enriched DNA from the immunoprecipitates (IPs) was quantified by RT-PCR using primers spanning the pri-miR-204 upstream regions. As shown in Figure 6G-J, enrichments of SNAI2, SUZ12, STAT3 and HDAC1 were confirmed at three different binding sites when comparing with the negative control (NEG) and decreased in cells treated with SNAI2, SUZ12, STAT3 and HDAC1 siRNAs, respectively. Similar results were observed in cells treated miR-204-5p mimics (Figure 6K-N). As expected, the enrichments of EZH2 and H3K27me3 in the miR-204-5p region were also strongly repressed in HNSCC cells treated with miR-204-5p mimics (Figure 6O-P). To characterize how the SNAI2/PRC2/HDAC1 complex and STAT3 functioned together in pri-miR-204 promoter at both of SNAI2 and STAT3 binding sites, ChIP experiments were further performed for SUZ12, SNAI2, STAT3, HDAC1 and H3K37me3 using crosslinked chromatin from HNSCC cells treated with SNAI2 and STAT3 siRNAs. As shown in Figure 6Q-R, the enrichments of SUZ12 were not significantly changed when SNAI2 and STAT3 were silenced. However, the enrichments of HDAC1, EZH2 and H3K27me3 were all significantly reduced by silencing the expression of SNAI2 and STAT3 expression (Figure 6S-X). On the other hand, the enrichments of SNAI2 and STAT3 were also dependent on each other (Figure 6Y-Z**)**. These data showed that STAT3 could enhance the enrichments of SNAI2, EZH2 and HDAC1 to the promoter of pri-miR-204 and then suppress its transcriptional activity. Collectively, these findings confirmed that the SNAI2/PRC2/HDAC1 complex and STAT3 suppresses miR-204-5p expression at transcriptional level, which in turn enhances recruitments of the SNAI2/PRC2/HDAC1 complex and STAT3 to the promoter of pri-miR-204. Moreover, our results confirmed that STAT3 could function as a gene suppressor by interacting with PRC2, SNAI2 and HDAC1.

### MiR-204-5p is inversely associated with its target genes and disease-free survival in HNSCC patients

**Figure 7:**
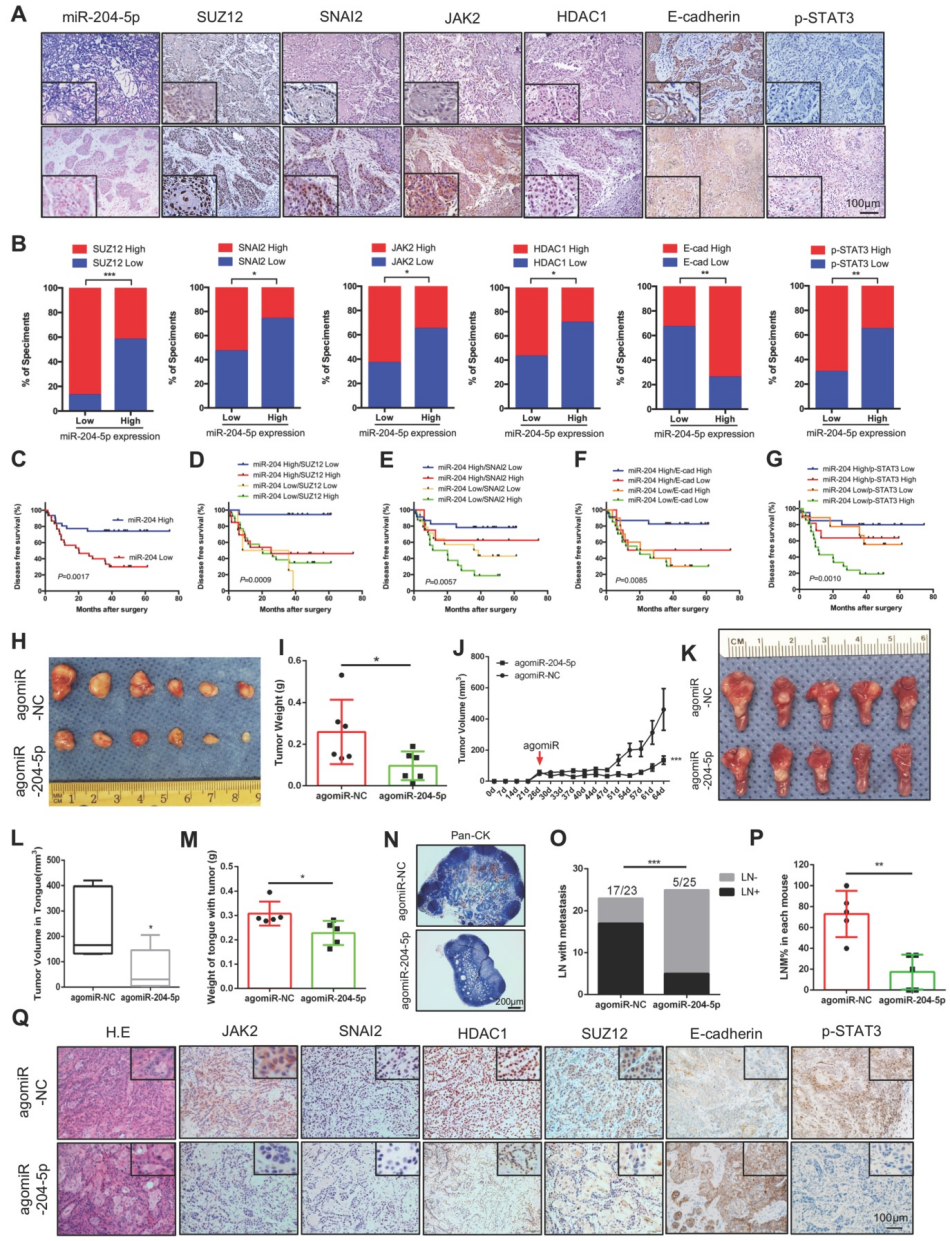
** Decrease of miR-204-5p indicates a poor prognosis and agomiR-204-5p inhibits tumor progression in PDX model.** (A) Representative image of ISH and IHC analysis of miR-204-5p, SUZ12, SNAI2, JAK2,HDAC1, E-cadherin, and p-STAT3 in HNSCC samples. (B) Percentage of specimens showing low or high miR-204-5p expression in relation to the expression levels of SUZ12, SNAI2, JAK2,HDAC1, E-cadherin, and p-STAT3.The Chi square test was used to analyze statistical significance. (C) Kaplan-Meier curves for survival of patients with HNSCC that grouped by the expression of miR-204-5p. Kaplan-Meier analyses for survival of patients with HNSCC that have expression levels of miR-204-5p high or low combined with SUZ12 (D), SNAI2 (E), E-cadherin (F), and p-STAT3 (G) high or low. *P* values of Kaplan-Meier analyses were calculated using log-rank and Gehan-Breslow-Wilcoxon tests. (H) Image of subcutaneous HNSCC PDX treated with agomiR-204-5p and agomiR-NC. Quantitative mass of tumors (I) and tumor growth kinetics (J) from HNSCC PDX treated with AgomiR-204-5p and agomiR-NC. Values are mean ± SD. n=6. *P < 0.05 by Student's t test. ***P < 0.001 by two-way ANOVA. (K) Image of orthotopic PDX treated with agomiR-204-5p and agomiR-NC. Quantitative of tumor volume (L) and tongue weight with tumor (M) from orthotopic PDX treated with agomiR-204-5p and agomiR-NC. (N) Representative image of pan-CK staining in lymph node. (O) The percentage of lymph node with metastatic tumor cells was analyzed by Fisher's exact test. ***P < 0.001. (P) The percentage of lymph node with metastatic tumor cells in each mouse. **P < 0.01 by Student's t test. (Q) Representative image of H.E and IHC staining for miR-204-5p related genes from subcutaneous HNSCC PDX treated with agomiR-204-5p and agomiR-NC. E-cad: E-cadherin.

Next, the clinical relevance of miR-204-5p and its targets were validated in an HNSCC patient cohort. As shown in Figure 7A-B, the expression of miR-204-5p was negatively associated with the protein expression levels of SUZ12, SNAI2, JAK2, HDAC1 and p-STAT3 but positively correlated with E-cadherin expression. A Kaplan-Meier analysis showed that low expression of miR-204-5p level is significantly associated with short disease-free survival (Figure 7C). As expected, we observed that high expression of SUZ12, SNAI2, p-STAT3 and low expression of E-cadherin indicated a poor prognosis in HNSCC patients (Figure S5A-D). Similar results were also observed in our previous studies 20, 22. However, the DFSs were not significantly changed in patients with high JAK2 and HDAC1 expression when comparing to those with low expression (Figure S5E-F). Importantly, we found that patients with both low miR-204-5p expression and high expression of related genes showed the poorest survival rate (Fig. 7D-G and Figure S5G-H). Thus, loss of miR-204-5p is associated with increased SUZ12, SNAI2, HDAC1, JAK2, p-STAT3 expression levels but decreased expression of E-cadherin, and their associations are clinically relevant. Collectively, these data support the assertion that miR-204-5p has key tumor suppressor functions in HNSCC that are largely determined by its ability to silence SUZ12, SNAI2, JAK2 and HDAC1, and thus inhibit clinically aggressive tumors.

### Administration of agomiR-204-5p inhibits tumor growth and metastasis in a HNSCC patient-derived xenograft model

To investigate whether miR-204-5p could serve as a potential therapeutic target, a HNSCC patient-derived xenograft (PDX) model was established and agomiR-204-5p was administrated by intra-tumoral and/or tail vein injection. After treatment with agomiR-204-5p, tumor growth was significantly suppressed, both the tumor weight and tumor volume were significantly decreased in mice treated with agomiR-204-5p when compared to control mice (Figure 7H-J). To further validate the therapeutic value of miR-204-5p, an orthotopic xenograft model was established by injecting the tumor cells to the floor of the tongue and treating animals with agomiR-204-5p. In line with the subcutaneous model, the tumor volume and weight of tongue with tumor were significantly decreased in mice bearing tumors treated in this way (Figure 7K-M). To evaluate the effect of agomiR-204-5p on cervical lymph node metastasis, neck dissection was performed to harvest all the cervical lymph nodes, including the submandibular lymph nodes, superficial cervical lymph nodes, facial lymph nodes and internal jugular lymph nodes. As shown in Figure S6A-B, the lymph node volumes were significantly reduced in tumor bearing mice treated with agomiR-204-5p. Microscopically, pan-CK staining revealed that the cervical lymph node metastasis was significantly inhibited by administration of agomiR-204-5p (Figure 7N-P). Consistently, staining of xenograft sections with IHC showed that expression of SUZ12, SNAI2, HDAC1, JAK2 and p-STAT3 was decreased but E-cadherin expression was increased in agomiR-204-5p-treated mice as compared to those in the control group (Figure 7Q), confirming that the administration of miR-204-5p could suppress tumor progression by targeting SUZ12, SNAI2, JAK2 and HDAC1 in vivo. Collectively, these data support the notion that miR-204-5p is a promising therapeutic target that can inhibit tumor growth and metastasis in HNSCC.

## Discussion

**Figure 8:**
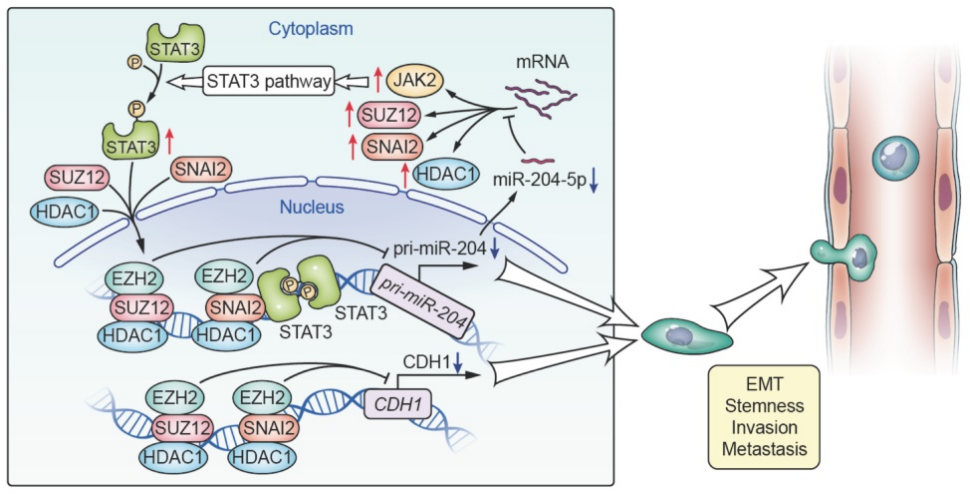
** Proposed model for miR-204-5p-SNAI2/SUZ12/HDAC1/JAK2/STAT3 regulatory circuit in HNSCC progression.** SNAI2, SUZ12, HDAC1 and JAK2 were all the direct targets of miR-204-5p. Loss of miR-204-5p promoted formation of a SNAI2/SUZ12/HDAC1 repressor complex and activated the JAK2/STAT3 signaling pathway, which in turn transcriptionally inhibited the expression of miR-204-5p and CDH1. miR-204-5p-SNAI2/SUZ12/HDAC1/STAT3 feedback loop is necessary to maintain a partial EMT phenotype in HNSCC.

The present study represents the most comprehensive investigations performed to date on the role of miR-204-5p in HNSCC. We uncovered a novel oncogenic pathway with broad biological and clinical relevance involving loss of miR-204-5p with activation of EMT and STAT3 signaling, which revealed the mechanisms governing tumor-initiating and metastatic properties and provided a novel therapeutic target for HNSCC. Notably, we identified that SNAI2, SUZ12, HDAC1 and JAK2 were all the direct targets of miR-204-5p, and found that loss of miR-204-5p promotes formation of a SNAI2/PRC2/HDAC1 repressor complex, which in turn inhibited the expression of miR-204-5p and CDH1 at transcriptional level. Also, we found that STAT3 could interact with SNAI2, PRC2 and HDAC1 to inhibit the expression of miR-204-5p at transcriptional level. These findings indicate that a miR-204-5p-SNAI2/SUZ12/HDAC1/STAT3 feedback loop is necessary to maintain a partial EMT phenotype and stemness in HNSCC (Figure 8). Furthermore, we showed that administration of ago-miR-204-5p impairs tumor growth and metastasis in HNSCC. These results provide mechanistic and translational insights into the tumorigenesis and metastasis of HNSCC and suggest that the miR-204-5p may serve as a promising therapeutic target for patients with HNSCC.

miRNAs are naturally occurring small non-coding RNA that regulate targeting gene expression at post-translational level 6, 7. Accumulating evidence suggests that the deregulation miRNA have a pivotal role in human cancers, including HNSCC 7, 10, 29. However, it is difficult to prioritize candidate miRNAs specific for cancer progression. In this study, we identified miR-204-5p to be one of the most important hub miRNAs in HNSCC by using an unbiased miRNA screening and Bayesian gene co-expression networks based on microarray analysis. In line with our findings, several studies reported that the expression of miR-204-5p was decreased in several different types of HNSCC based on different approaches, indicating that miR-204-5p might have a critical suppressive role in HNSCC 30-32. Then, our clinical and *in vivo* studies highlighted the tumor suppressive role of miR-204-5p in HNSCC progression, which was also confirmed in several other solid tumors, including neuroblastoma 33, lung cancer 34, gastric cancer 35, colorectal cancer 36 and glioma 37, implicating dysregulated miR-204-5p as a common event across various cancer types. However, the upstream and downstream molecular mechanisms of miR-204-5p in HNSCC are still not well documented in HNSCC.

Our previous studies suggested that change in miRNA-associated phenotypes could be mediated by a single miRNA target in HNSCC 17, 38, 39. However, miRNAs are well-known to regulate multiple target genes at post-transcription level and some miR-204-5p target genes have been identified in HNSCC, including IGFBP5 40, CXCR4 41, FOXC1 42, SNAI2 and SOX4 43. It remains a challenge to identify the key biological process and target genes perturbed by miRNAs in the context of cancer. To elucidate the effect of miR-204-5p on HNSCC progression, RNA-seq was performed and we found that EMT and stemness are most significantly changed in cancer cells treated with miR-204-5p mimics. In line with our findings, similar results were also observed in other tumor types 35, 37.

Next, we identified SNAI2, SUZ12, HDAC1 and JAK2 as being direct targets of miR-204-5p by utilizing unbiased approaches that were involved in EMT and stemness. SNAI2 is a well-known EMT transcription factor and JAK2 is the classic kinase involved in STAT3 signaling. Both have been identified as the direct target of miR-204-5p 43, 44. These findings indicated that miR-204-5p could suppress EMT and properties of cancer stem cells. Importantly, we identified two novel new targets for miR-204-5p including HDAC1 and SUZ12, a subunit of PRC2, which were responsible for histone modification and involved in regulating EMT and stemness 45, 46. Strikingly, we further found that SNAI2, SUZ12 and HDAC1 could form a repressor complex to regulate target gene expressions at transcriptional levels. Consistently, EZH2, another PRC2 subunit, has been confirmed to interact with HDAC1/HDAC2 and SNAI1 to form a complex to repress E-cadherin in nasopharyngeal carcinoma 24. These findings support the hypothesis that the interaction of PRC2 with members of the SNAIL family is a universal mechanism involving in cancer progression.

However, our ChIP-PCR results showed that the enrichments of SUZ12 at the promoter region of CDH1 and pri-miR-204 were not significantly changed by modulating SNAI2 and STAT3 expression, indicating that SUZ12 may be pre-bound or recruited by other factors. It has been found that SNAI1 recruits SUZ12 and EZH2 to the promoter of CDH1 and represses transcription with a higher efficiency and potency than SNAI2 47-49. We showed that SNAI2 could recruit EZH2 but not SUZ12, suggesting SNAI1 and SNAI2 have different functions and non-overlapping roles in cancer cells and might cooperate to recruit PRC2 to the CDH1 promoter. To address the role of pre-bound SUZ12 in the CDH1 promoter, we knocked down SUZ12 with siRNAs and found that EZH2 and H3K27me3 enrichments were reduced, confirming that SUZ12 was critical for EZH2 recruitment and H3K27 tri-methylation. Interestingly, recent studies showed that SUZ12 could interact with all subunits of PCR2 to maintain the stability of the PRC2 complex and bind CpG islands (CGIs) 50, 51. These results implied that pre-bound SUZ12, through recognizing CGIs at target genes, was critical to direct PRC2 and maintain H3K27me3 patterns. On the other hand, we showed that SNAI2 recruits HDAC1 to the promoter of the CDH1 gene and maintains local histone deacetylation. Thus, these findings reveal a novel mechanism of governing the EMT phenotype in HNSCC, in which SNAI2, PRC2 and HDAC1 coordinately suppress CDH1 transcriptional activity by forming a repressive complex at both transcriptional and epigenetic levels. Importantly, this transcription repression can be abrogated by restoration of miR-204-5p expression both *in vitro* and* in vivo.*

Since, as our data indicated, the loss of miR-204-5p is a frequent and key event in HNSCC progression, we further investigated the mechanisms involved in the repression of miR-204-5p expression. As is well known, miR-204-5p is located within the sixth intron of the host gene transient receptor potential melastatin 3 (TRPM3) cation channel. It is transcribed in the same direction as TRPM3, which is located on human chromosome 9q21.11, and exhibits a high frequency of loss of heterozygosity (LOH) in human HNSCC 30. Generally, LOH at 9q21.1-q22.3 occurs in 37% of premalignant head and neck lesions, and increases to 67% in HNSCC 52, 53. However, our data showed that the expression of miR-204-5p was decreased in 89.66% of patients with HNSCC compared with paired adjacent non-cancerous counterparts. This suggests that the expression of miR-204-5p might be regulated by other genetic and epigenetic mechanisms.

Interestingly, we found two E-boxes located 681bp upstream and 15bp downstream of the pri-miR-204 transcription stating site (TSS), and one E-box located 223bp downstream of TRPM3 TSS, which is a well-known binding site for SNAI2. This indicates that the novel SNAI2/PRC2/HDAC1 complex might be involved in regulating the expression of miR-204-5p through a similar mechanism as presence on CDH1 promoter, which was confirmed by qPCR and ChIP assay. These findings establish a novel regulatory circuit between miR-204-5p and the SNAI2/PRC2/HDAC1 repressive complex. In addition, the STAT3 binding sites were also observed in the promoter region of pri-miR-204 and TRPM3 close to the E-box. Of note, the direct suppression of miR-204-5p by STAT3 was confirmed by qPCR and ChIP. Consistent with these data, the suppressive role of STAT3 was previously reported in endometrial carcinoma 54. However, the exact mechanism of STAT3-mediated inhibition of miR-204-5p transcription is still not clear. Our results reveal that STAT3 regulates the enrichment of SNAI2 and recruits HDAC1 and PRC2 to the promoter of pri-miR-204, followed by modifying H3K27 tri-methylation to repress the transcription. Furthermore, it has been confirmed that the interaction of PRC2 and STAT3 enhances STAT3 activity by increasing tyrosine phosphorylation of STAT3, and the activation of STAT3 signaling in turn promotes SNAI2 expression 55-57. Altogether, these results confirmed that the interaction between the SNAI2/PRC2/HDAC1 repressive complex and STAT3 is associated with miR-204-5p mediated phenotype changes and its suppression. To support this regulatory circuit, the negative correlation is observed between miR-204-5p expression and SNAI2, SUZ12, HDAC1, JAK2 and p-STAT3 expression, and patients with low expression of miR-204-5p and high expression of its targets showed the poorest prognosis in HNSCC. Our results, therefore, also have important therapeutic implications in that restoration of miR-204-5p might be highly effective in treatment of HNSCC with minimal side effects. To this end, we showed that the agomiR-204-5p could inhibit tumor growth and metastasis in an HNSCC PDX model with repression of EMT-mediated by the SNAI2/PRC2/HDAC1 complex and inactivation of JAK2/STAT3 signaling; all of which indicates that targeting miR-204-5p is a promising and innovative therapeutic strategy. However, targeted delivery of miRNA-204-5p to the cancer cells is still a challenge for miRNA-based treatment in HNSCC. It's urgent to develop a novel delivery system which might provide a better treatment outcome with a less side effect, such as nanoparticles 58 and polyamidoamine dendrimer 59.

In summary, our data identify a key oncogenic regulatory circuit by loss of miR-204-5p leading to activation of JAK2/STAT3 signaling and partial EMT program via directly targeting JAK2 and the SNAI2/SUZ12/HDAC1 repressive complex. In turn, the SNAI2/SUZ12/HDAC1 repressive complex and STAT3 cooperatively suppress the transcription of miR-204-5p. These findings uncover a novel molecular mechanism that maintains the partial EMT phenotype and the properties of tumor initiating cells and may be seen as a prognostic biomarker and therapeutic target for HNSCC.

## Methods and Materials

### Cell culture

Human HNSCC cell lines SCC9, SCC15, SCC25, CAL27 and HEK293T cells were obtained from ATCC (Rockville, MD, USA). UM1 and UM-SCC1 was provided by Dr. Xiaofeng Zhou (University of Illinois at Chicago, IL, USA). HSC3, HSC6 and CAL33 were kindly provided by J. Silvio Gutkind (NIH, Bethesda, MD, USA). The characteristics of HNSCC cell lines are described in Table S1. The UM-SCC1, HSC3, HSC6, CAL27, CAL33 and HEK293T cells were cultivated in Dulbecco's modified Eagle's medium (DMEM, Gibco, Rockville, MD, USA) supplemented with 10% fetal bovine serum (FBS, Gibco). The SCC9, SCC15, SCC25 and UM1 cells were maintained in DMEM-F12 (Gibco) supplemented with 10% FBS. All cells were incubated at 37°C in a humidified atmosphere containing 5% CO_2_. All cell lines were routinely tested for Mycoplasma by PlasmoTest^TM^ Mycoplasma contamination detection kit (InvivoGen).

### Patients and tissue specimens

A total of 61 fresh and paraffin-embedded human HNSCC specimens were enrolled in this study, which were pathologically diagnosed at the Hospital of Stomatolgoy, Sun Yat-sen University from 2012 to 2015. The clinicopathological features of the HNSCC patients are described in Table S2. All patients received radical surgery. None received any form of pre-surgical adjuvant therapy. Clinicopathological staging of the tumor was determined according to the TNM classification system of UICC. Survival time was calculated from the date of surgery to the date of the final follow-up or cancer recurrence. The date of death was obtained from the medical records or follow-up telephone calls. The normal oral epithelium/tissue was harvested from the surgical removed specimen far from the cancer tissue. All the clinical specimens were collected after approved by the Ethical Committee of Hospital of Stomatology, Sun Yat-sen University, and written informed consent was obtained from all participants or their appropriate surrogates. All patients' specimens used in this study were conducted in accordance with the Declaration of Helsinki.

### Animal studies

6-week-old female BALB/c nude mice used for this study were purchased from the Animal Care Unit of Guangdong, China and maintained in pathogen-free conditions. All animal studies were conducted with the approval of the Sun Yat-sen University Institutional Animal Care and Use Committee and were performed in accordance with established guidelines. For subcutaneous xenograft model, UM-SCC1 cells stably overexpressing miR-204-5p (Lenti-204) or negative control (Lenti-ctrl.) were harvested, washed, counted, and resuspended in a 1:1 solution of DMEM-F12 and Matrigel (Corning, NY, USA). Then the nude mice were injected subcutaneously in the flanks with indicated cells. For orthotopic xenograft model, the indicated cells were injected into the tongue of nude mice. Tumor volume (mm^3^) was measured using a caliper at indicated time, and calculated using the following formula: V=(length×width^2^)/2. The mice were sacrificed at the indicated endpoint after injection. The tumors were removed and weighed. The lymph nodes were harvested and assessed in the orthotopic xenograft model. For *in vivo* metastasis assay, indicated cells were injected into each nude mouse by tail vein. The mice were sacrificed and lung and liver metastases were evaluated when one of them became moribund. For *in vivo* agomiR-204-5p administration, the subcutaneous and orthotopic patient derived xenograft model were established. The miR-204-5p agomiR (ago-miR-204-5p, RiboBio) or control agomir (ago-miR-NC) was administrated by intra-tumoral or tail vein injection 3 times per week for 4 weeks when the average volume of xenograft tumors reached approximately 50 mm^3^. Tumor growth was routinely observed. Mice were sacrificed and tumors were then collected, weighted, Fixed and paraffin-embedded.

### Luciferase reporter assay

To generate HNSCC stable STAT3 luciferase reporter cell lines, UM-SCC1 or UM-1 cell were seeded in 10 cm dishes and incubated overnight. The next day cells were treated with STAT3 luciferase reporter lenti viral particles (Cellomics Technology, Halethorpe, MD, USA). After 12 h, the medium was replaced with fresh medium. From the next day onward, the cells were selected using puromycin (2 mg/ml) for at least 3-4 days. For luciferase assays, 48 h post miRNA transfection, the luciferase activity of total cell lysates was measured using Bright-Glo™ Luciferase Assay System (Promega, Madison, WI, USA). The STAT3 luciferase activity was normalized against the protein concentration of each cell lysate sample. To generate the 3'-UTR reporter construct of target genes, the 3'-UTRs of SUZ12, SNAI2, JAK2, and HDAC1, respectively, were amplified and cloned downstream to the luciferase reporter in the pmiR-GLO vector (Promega, Madison, WI, USA). The reporter plasmids containing wild-type (AATGAAGGGA for SUZ12; TTTAAAAGGGA for SNAI2; ACATAAAGGGA for JAK2; GGTGAAAGGGA for HDAC1) or mutated (AATGGGAAAG for SUZ12; TTTATCTCTCT for SNAI2; ACATTCTCTCT for JAK2; GGTGTCTCTCT for HDAC1) miR-204-5p binding sites were designed and synthesized by GenePharma (Shanghai, China). The reporter plasmids were transfected into cell lines using the Lipofectamine® 3000 Transfection Reagent (Invitrogen), according to the manufacturer's recommendation. Luciferase reporter assays were performed 48h after the transfections using the Dual-Luciferase Reporter Assay System (Promega) as the manufacturer's instructions. Measurements from triplicate transfections were expressed as relative luciferase activity corrected for Renilla luciferase activity and normalized to the control.

### IP, RIP-PCR and ChIP-PCR assay

Cells were lysed in Cellytic buffer (Sigma-Aldrich) for 5 min on ice. After centrifugation at 12,000 g at 4 °C for 10 min, the supernatants were incubated with antibodies at 4 °C overnight, followed by incubation with protein A or protein G Dynabeads (Invitrogen) for additional 2 h at 4 °C. Immunoprecipitates were washed three times with PBS and 0.1% NP40 buffer. Proteins bound to the beads were eluted with SDS loading buffer at 98 °C for 5 min and then subjected to SDS-PAGE. For ChIP-qPCR assays, cells were treated with 10 mM dimethyl 3,30-dithiobispropionimidate-HCl (DTBP) (Pierce, Rockford, IL, USA) in PBS for 10 min at room temperature, and then treated with 1% formaldehyde at 37 °C for 10 min. Total cell lysates were sonicated to generated 400-600 bp DNA fragments. For each ChIP reaction mixture, 10^6^ cells were used. IP was performed as described previously 60. The resulting precipitated DNA samples were quantified by quantitative real-time PCR (qPCR). Data are expressed as the percentage of input DNA. The primer sequences used for ChIP-qPCR were as listed in Table S3. For RIP-qPCR, cells were either transfected with miR-204-5p mimics or control mimics. Two days post transfection, immunoprecipitation was performed following the protocol described for EZ-Magna RIP™ RNA-Binding Protein Immunoprecipitation Kit (Millipore, Billerica, MA, USA). RNA that co-immunoprecipitated with anti-AGO2 antibodies was extracted and qRT-PCR was performed. The primer sequences used for RIP-qPCR were as listed in Table S3. The antibodies used for IP, RIP-PCR and ChIP-PCR assay were as listed in Table S4.

### Plasmid and generation of stably engineered cell lines

UM1 or UM-SCC1 cells with stable expression of miR-204-5p (Lenti-204) and an empty vector as control (Lenti-Ctrl) were established by infection with the pEZX-MR03 lentiviral vector (GeneCopoeia, Rockville, MD, USA). The Lenti-204 plasmid encoded the human genomic pre-miR-204 gene, which was sequence-verified. To establish stable cell lines, the lentiviral vectors were packaged and replicated in HEK293T cells. Viruses were harvested at 48 h after transfection, filtered, and used for infection of UM1 or UM-SCC1 cells in the presence of 5 μg/mL polybrene (Sigma-Aldrich, St Louis, MO, USA). After infection, puromycin (0.5 μg/mL, Sigma-Aldrich) was used to select stably transduced cells over 2 weeks. qRT-PCR was performed to verify miR-204-5p expression in all cells used for these experiments.

### RNA extraction, reverse transcription, and real-time RT-PCR

Total RNA was isolated from cells using the miRNeasy Mini Kit (QIAGEN, Duesseldorf, Germany). To detect the miRNAs levels, cDNA was synthesized from total RNA and analyzed by qRT-PCR using the Bulgeloop™ miRNA qRT-PCR Primer Sets (RiboBio, Guangzhou, China) and SYBR GREEN I Master Mix (Roche, Basel, Switzerland). To determine the pri-miR-204 and mRNA levels, reverse transcription was performed using the Transcriptor First Strand cDNA Synthesis Kit (Roche). For pri-miR-204 expression, the cDNA was subjected to TaqMan Probe-based RT-qPCR (Light Cycler 480 Probes Master, Roche) with specific primers (TaqMan Pri-miRNA Assays ID: Hs03293531_pri, Applied Biosystems, Foster City, CA, USA). The expression was normalized to GAPDH (TaqMan Gene Expression Assays ID: Hs03929097_g1, Applied Biosystems). Detection of mRNA was performed as described previously 61. Primer sets used for qRT-PCR were synthesized by Invitrogen (Carlsbad, CA, USA) and the sequences were as listed in Table S3. The gene relative expression levels above were calculated using the 2^- ΔΔCt^ method after normalization to the GAPDH or U6.

### Western blot

Western blot was performed according a standard method as described previously 17, 21, 22, 61. The primary antibodies were as listed in Table S4.

### Immunofluorescence

Immunofluorescence was performed as described previously 38. In brief, cells were transfected with miR-204-5p mimics or inhibitors and then fixed with 4% para-formaldehyde, permeabilized with 0.5% Triton X-100, blocked with 1% BSA in PBS, and subsequently incubated with primary antibodies. The primary antibodies were as listed in Table S4. The cells were then incubated with Alexa Fluor 488-conjugated anti-rabbit IgG antibody (#4412, Cell Signaling Technology, Danvers, MA, USA) and Alexa Fluor 594-conjugated anti-rabbit IgG antibody (#8889, Cell Signaling Technology) at room temperature for one hours in the dark. The cells then washed with PBS and counterstained with DAPI (Invitrogen) for 5 min following the manufacturer's protocol. Images were obtained with a laser scanning microscope (Carl Zeiss, Jena, Germany).

### Sphere formation assay, CCK8, clonogenic formation assay and ALDEURF assay

For sphere formation assays, cells were plated in serum-free DMEM/F12 medium supplemented with 2% B27 (Gibco), 1% N2 (Gibco), 20 ng/mL EGF (Invitrogen), 10ng/mL bFGF (Invitrogen) and 4ug/mL insulin (Gibco) in ultra-low attachment plate. The number and diameters of spheres with a diameter over 40μm were counted and measured 1 week after plating by a microscopy (Carl Zeiss).

Cell proliferation was analyzed using the Cell Counting Kit-8 (CCK-8, Sigma-Aldrich). Briefly, 2×10^3^ cells were seeded in triplicate into a 96-well plate. Cell viability was assessed at 1, 2, 3 and 4-day post-transfection. The absorbance was measured at 450 nm using a microplate reader (Genios TECAN, Männedorf, Schweiz).

For colony formation assays, 5×10^2^ cells were seeded into 6-well plates. After culturing for 10 days, visible colonies were stained with crystal violet. Colonies with diameters above 1 mm were counted.

The ALDH activity was measured by using ALDEFLUOR™ detection kit (STEMCELL Technologies, Vancouver, BC, Canada) according to the manufacturer's instructions and data were acquired on a CytoFLEX (Beckman Coulter, Miami, FL, USA).

### Wound healing, cell migration and invasion assays

The *in vitro* cell migration and invasion were measured using the BD BioCoat™ system (BD Biosciences, San Jose, CA, USA) following the manufacturer's instructions. In brief, cells were seeded in the upper chambers, and culture medium with 10% FBS was added to the lower chambers. For invasion assay, transwell inserts coated with Matrigel were used. After 24 h or 48 h incubation, cells that migrated to the reverse side of inserts were stained with Diff-Quik stain kit (Sysmex, Kobe, Japan) and quantified.

An *in vitro* wound healing assay was used to evaluate cell motility 62. In brief, cells were seeded to 6-well plate and allowed to grow to confluence. A wound was scratched in the cell layer with a sterile micropipette tip. Then, the cell layer was gently washed twice with medium to remove the detached cells and incubated for 48 h in medium with 1% FBS. The size of the scratch was measured at 5 random sites in photographs taken using a camera (Carl Zeiss).

### Transient transfection and drug treatment

Cells were transfected with miRIDIAN microRNA has-miR-204-5p mimics or inhibitors or corresponding control oligos (Dharmacon, Lafayette, CO, USA), or SNAI2, SUZ12, HDAC1, STAT3 siRNA or control siRNA (ON-TARGETplus SMARTpool siRNA, Dharmacon,) by using Lipofectamine RNAiMax (Invitrogen) as per the manufacturer's instructions. For plasmid transfection, Lipofectamine® 3000 Transfection Reagent (Invitrogen) was used according to manufacturer's instructions. After culturing for 48 h, transfected cells were harvested for the following studies. For drug treatment, cell lines were treated for 24 h with the following inhibitors: EZH2 inhibitor GSK126 (Cat#S7061, Selleck Chemicals, Houston, TX, USA) at 5 μM and HDAC inhibitor Vorinostat (suberoylanilide hydroxamic acid, SAHA, Cat#S1047, Selleck Chemicals) at 3 μM.

### Immunohistochemistry and in situ hybridization assay

Immunohistochemistry assays were performed and quantified as previously described 61. Formalin-fixed, paraffin-embedded tumor tissues were cut into 4 μm sections and processed for immunostaining. The sections were incubated overnight at 4 °C with primary antibodies. The slides were then incubated with horseradish perioxidase-labeled polymer for 60 min. The immunocomplexes were detected with AEC+ chromogen (Dako, Carpinteria, CA, USA) and counterstained with hematoxylin. The degree of immunostaining of indicated proteins was assessed and scored by two senior pathologists who were blinded to the clinical data. The staining intensity of the cells was recorded as follows: 0 (no staining), 1 (weak, light yellow), 2 (moderate, yellow brown) and 3 (strong, brown). The proportion of positively stained cells was graded as follows: 0 (0%), 1 (<10%), 2 (<50%), 3 (<75%), 4 (≥75%). The staining index (SI) was calculated according to both the staining intensity and the proportion of positive staining tumor cells per the following formula: SI= the proportion of positively stained cells × the staining intensity, which resulting in scores of 0, 1, 2, 3, 4, 6, 8, 9, and 12. Cases with SI≥6 were considered to be high expression, and those with SI <6 were considered to be low expression.

MiR-204-5p expression was examined using in situ hybridization (ISH) in tissue sample as the manufacturer's protocol (microRNA ISH Optimization Kit for FFPE, Exiqon, Vedbaek, Denmark). Briefly, after demasking, miR-204-5p was hybridized with Double-DIG-labeled LNA™ microRNA probes (1:000, Exiqon) overnight, subsequently incubated overnight with an anti-DIG monoclonal antibody (Roche). The sections were counterstained with nitro blue tetrazolium/5-bromo-4-chloro-3-indolylphosphate (NBT/BCIP, Roche). The staining scores were determined based on both the intensity and proportion of the miR-204-5p-positive cells (in blue). The proportion of positively stained cells was graded as described above for immunohistochemistry assay. The staining intensity of the cells was recorded as follows: 0 (no staining), 1 (weak, light blue), 2 (moderate, blue) and 3 (strong, dark blue). The staining index (SI) was calculated as SI=staining intensity × proportion of positively stained cells. SI (miR-204-5p) ≥6 was defined as high expression, and SI<6 was defined as low expression.

### miRNA microarray, RNA-seq and bioinformatics analysis

For miRNA microarray, total RNA was amplified, labeled and purified by an Affymetrix WT PLUS Reagent Kit (Affymetrix, Santa Clara, CA, USA) according to the manufacturer's instructions to obtain biotin-labeled cDNA. Arrays were scanned with an Affymetrix GeneChip® Scanner 3000 (Affymetrix). Command Console Software (Affymetrix) was used to control the scanner and summarize probe cell intensity data (CEL file generation) with default settings. The raw data were normalized by an Expression Console. The 'GRENITS' package from R Bioconductor was used to cluster co-miRNA into causal interaction networks from miRNA microarray data in groups of HNSCC and normal tissues using the fully Bayesian spline autoregression 63. Differential 51 miRNA with interaction network probability > 0.1 or >0.5 in disease groups or normal groups were filtered for further function-associated network analysis using Cytoscape software, respectively.

For RNA-seq, Quality of the RNA for sequencing was determined using an Agilent 2100 Bioanalyzer (Agilent Technologies, Santa Clara, CA, USA). Library preparation was performed by using the Illumina TruSeq RNA Sample Pre Kit, and RNAs were sequenced on Illumina Hiseq Xten machines. The raw sequencing reads were received in FASTQ formatted file. The expression level of each annotated genes were calculated and differential expression (DE) analysis was performed by using edgeR.

For Gene Ontology analysis, IPA (QIAGEN) and GSEA (Broad Institute, Cambridge, MA, USA) were performed. We followed the standard procedure as described by GSEA user guide (http://www.broadinstitute.org/gsea/doc/GSEAUserGuideFrame.html). Gene expression profiles from RNA-seq were used to conduct GSEA to identify gene signatures between cells treated with miR-204-5p mimics or control mimics. We used a customized database collection of gene targets of PRC2 and H3K27 bound from Ben-Porath's study 64 and targets of SNAI2 form Mistry's study 65, and the GSEA algorithm (http://software.broadinstitute.org/gsea/index.jsp) to rank significant differential expression genes. GSEA results are shown using normalized enrichment scores.

The targets of miR-204-5p were predicted based on Starbase V3.0 (http://starbase.sysu.edu.cn/) 66.

STRING networks (https://stringdb.org/) were used for visualization of protein-protein interaction (PPI) among the target genes.

The TCGA Head and Neck Squamous Cell Carcinoma database for miRNA gene expression quantification data and clinical follow up data are analyzed by using TCGAbiolinks R package. TCGA-HNSCC miRNA expression data for all patients were normalized and filtered using TCGAbiolinks, then exported to Microsoft EXCEL.

### Statistics

Statistical analyses were performed as described in the figure legend for each experiment using Graphpad Prism 6.0. All data are presented as mean ± SD unless otherwise noted in the figure legend. All statistical tests were two-side and differences were considered statistically significant at *P* < 0.05. Limiting dilutions were calculated using the Extreme Limiting Dilution Analysis software (http://bioinf.wehi.edu.au/software/elda/).

### Data availability

The miRNA microarray data and RNA-Seq data discussed in this publication have been deposited in NCBI Gene Expression Omnibus with the accession number GSE124566 and GSE128773. All other relevant data are available from the corresponding authors upon reasonable request.

## Supplementary Material

Supplementary figures and tables.

Supplementary table of genes upregulated by FDR<0.05 in UM-SCC1 cells over-expressing miR-204-5p in RNA-Seq analysis.

## Abbreviations

HNSCC: head and neck squamous cell carcinoma
miR-204-5p: microRNA-204-5p
EMT: epithelial-mesenchymal transition
3' URT: 3' untranslated region
LNM: lymph node metastasis
IP: Immunoprecipitation
RIP: RNA immunoprecipitation
ChIP: Chromatin immunoprecipitation
NATs: non-cancerous adjacent tissues
TCGA: The Cancer Genome Atlas
ISH: In situ hybridization
qPCR: quantitative polymerase chain reaction
DEGs: differentially expressed genes
IPA: ingenuity pathway analysis
GSEA: Gene Set Enrichment Analysis
RISC: RNA-induced silencing complex
DFS: disease-free survival
PDX: patient-derived xenograft
IHC: immunohistochemistry
LOH: loss of heterozygosity
TSS: transcription stating site

## References

[1] Chinn SB, Myers JN (2015). Oral cavity carcinoma: current management, controversies, and future directions. J Clin Oncol.

[2] Sacco AG, Cohen EE (2015). Current treatment options for recurrent or metastatic head and neck squamous cell carcinoma. J Clin Oncol.

[3] Argiris A, Karamouzis MV, Raben D, Ferris RL (2008). Head and neck cancer. Lancet.

[4] Hedberg ML, Goh G, Chiosea SI, Bauman JE, Freilino ML, Zeng Y (2016). Genetic landscape of metastatic and recurrent head and neck squamous cell carcinoma. J Clin Invest.

[5] Bernier J, Domenge C, Ozsahin M, Matuszewska K, Lefebvre JL, Greiner RH (2004). Postoperative irradiation with or without concomitant chemotherapy for locally advanced head and neck cancer. N Engl J Med.

[6] Bartel DP (2004). MicroRNAs: genomics, biogenesis, mechanism, and function. Cell.

[7] Lin S, Gregory RI (2015). MicroRNA biogenesis pathways in cancer. Nat Rev Cancer.

[8] Kasinski AL, Slack FJ (2011). Epigenetics and genetics. MicroRNAs en route to the clinic: progress in validating and targeting microRNAs for cancer therapy. Nat Rev Cancer.

[9] Domingues C, Serambeque BP, Laranjo Candido MS, Marto CMM, Veiga FJB, Sarmento Antunes Cruz Ribeiro AB (2018). Epithelial-mesenchymal transition and microRNAs: challenges and future perspectives in oral cancer. Head Neck.

[10] Koshizuka K, Hanazawa T, Arai T, Okato A, Kikkawa N, Seki N (2017). Involvement of aberrantly expressed microRNAs in the pathogenesis of head and neck squamous cell carcinoma. Cancer Metastasis Rev.

[11] Karatas OF, Oner M, Abay A, Diyapoglu A (2017). MicroRNAs in human tongue squamous cell carcinoma: from pathogenesis to therapeutic implications. Oral Oncol.

[12] Koshizuka K, Hanazawa T, Fukumoto I, Kikkawa N, Okamoto Y, Seki N (2017). The microRNA signatures: aberrantly expressed microRNAs in head and neck squamous cell carcinoma. J Hum Genet.

[13] Zhao Z, Li L, Du P, Ma L, Zhang W, Zheng L (2019). Transcriptional downregulation of miR-4306 serves as a new therapeutic target for triple negative breast cancer. Theranostics.

[14] Ma M, Dai J, Tang H, Xu T, Yu S, Si L (2019). MicroRNA-23a-3p inhibits mucosal melanoma growth and progression through targeting adenylate cyclase 1 and attenuating cAMP and MAPK pathways. Theranostics.

[15] Li J, Huang H, Sun L, Yang M, Pan C, Chen W (2009). MiR-21 indicates poor prognosis in tongue squamous cell carcinomas as an apoptosis inhibitor. Clin Cancer Res.

[16] Chen Z, Jin Y, Yu D, Wang A, Mahjabeen I, Wang C (2012). Down-regulation of the microRNA-99 family members in head and neck squamous cell carcinoma. Oral Oncol.

[17] Xie N, Wang C, Zhuang Z, Hou J, Liu X, Wu Y (2016). Decreased miR-320a promotes invasion and metastasis of tumor budding cells in tongue squamous cell carcinoma. Oncotarget.

[18] Xie N, Wang C, Liu X, Li R, Hou J, Chen X (2015). Tumor budding correlates with occult cervical lymph node metastasis and poor prognosis in clinical early-stage tongue squamous cell carcinoma. J Oral Pathol Med.

[19] Wang C, Huang H, Huang Z, Wang A, Chen X, Huang L (2011). Tumor budding correlates with poor prognosis and epithelial-mesenchymal transition in tongue squamous cell carcinoma. J Oral Pathol Med.

[20] Puram SV, Tirosh I, Parikh AS, Patel AP, Yizhak K, Gillespie S (2017). Single-cell transcriptomic analysis of primary and metastatic tumor ecosystems in head and neck cancer. Cell.

[21] Wang C, Liu X, Huang H, Ma H, Cai W, Hou J (2012). Deregulation of Snai2 is associated with metastasis and poor prognosis in tongue squamous cell carcinoma. Int J Cancer.

[22] Wang C, Liu X, Chen Z, Huang H, Jin Y, Kolokythas A (2013). Polycomb group protein EZH2-mediated E-cadherin repression promotes metastasis of oral tongue squamous cell carcinoma. Mol Carcinog.

[23] Chang JW, Gwak SY, Shim GA, Liu L, Lim YC, Kim JM (2016). EZH2 is associated with poor prognosis in head-and-neck squamous cell carcinoma via regulating the epithelial-to-mesenchymal transition and chemosensitivity. Oral Oncol.

[24] Tong ZT, Cai MY, Wang XG, Kong LL, Mai SJ, Liu YH (2012). EZH2 supports nasopharyngeal carcinoma cell aggressiveness by forming a co-repressor complex with HDAC1/HDAC2 and Snail to inhibit E-cadherin. Oncogene.

[25] Lin CW, Wang LK, Wang SP, Chang YL, Wu YY, Chen HY (2016). Daxx inhibits hypoxia-induced lung cancer cell metastasis by suppressing the HIF-1alpha/HDAC1/Slug axis. Nat Commun.

[26] Tellez CS, Juri DE, Do K, Picchi MA, Wang T, Liu G (2016). MiR-196b is epigenetically silenced during the premalignant stage of lung carcinogenesis. Cancer Res.

[27] Au SL, Wong CC, Lee JM, Fan DN, Tsang FH, Ng IO (2012). Enhancer of zeste homolog 2 epigenetically silences multiple tumor suppressor microRNAs to promote liver cancer metastasis. Hepatology.

[28] Ryu S, McDonnell K, Choi H, Gao D, Hahn M, Joshi N (2013). Suppression of miRNA-708 by polycomb group promotes metastases by calcium-induced cell migration. Cancer cell.

[29] Lai YH, Liu H, Chiang WF, Chen TW, Chu LJ, Yu JS (2018). MiR-31-5p-ACOX1 axis enhances tumorigenic fitness in oral squamous cell carcinoma via the promigratory prostaglandin E2. Theranostics.

[30] Lee Y, Yang X, Huang Y, Fan H, Zhang Q, Wu Y (2010). Network modeling identifies molecular functions targeted by miR-204 to suppress head and neck tumor metastasis. PLoS Comput Biol.

[31] Lapa RML, Barros-Filho MC, Marchi FA, Domingues MAC, de Carvalho GB, Drigo SA (2019). Integrated miRNA and mRNA expression analysis uncovers drug targets in laryngeal squamous cell carcinoma patients. Oral Oncol.

[32] Pedersen NJ, Jensen DH, Lelkaitis G, Kiss K, Charabi BW, Ullum H (2018). MicroRNA-based classifiers for diagnosis of oral cavity squamous cell carcinoma in tissue and plasma. Oral Oncol.

[33] Ooi CY, Carter DR, Liu B, Mayoh C, Beckers A, Lalwani A (2018). Network modeling of microRNA-mRNA interactions in neuroblastoma tumorigenesis identifies miR-204 as a direct inhibitor of MYCN. Cancer Res.

[34] Xia Y, Zhu Y, Ma T, Pan C, Wang J, He Z (2014). MiR-204 functions as a tumor suppressor by regulating SIX1 in NSCLC. FEBS Lett.

[35] Zhang L, Wang X, Chen P (2013). MiR-204 down regulates SIRT1 and reverts SIRT1-induced epithelial-mesenchymal transition, anoikis resistance and invasion in gastric cancer cells. BMC cancer.

[36] Yin Y, Zhang B, Wang W, Fei B, Quan C, Zhang J (2014). MiR-204-5p inhibits proliferation and invasion and enhances chemotherapeutic sensitivity of colorectal cancer cells by downregulating RAB22A. Clin Cancer Res.

[37] Ying Z, Li Y, Wu J, Zhu X, Yang Y, Tian H (2013). Loss of miR-204 expression enhances glioma migration and stem cell-like phenotype. Cancer Res.

[38] Jin Y, Wang C, Liu X, Mu W, Chen Z, Yu D (2011). Molecular characterization of the microRNA-138-Fos-like antigen 1 (FOSL1) regulatory module in squamous cell carcinoma. J Biol Chem.

[39] Zhuang Z, Hu F, Hu J, Wang C, Hou J, Yu Z (2017). MicroRNA-218 promotes cisplatin resistance in oral cancer via the PPP2R5A/Wnt signaling pathway. Oncol Rep.

[40] Liu L, Wang J, Li X, Ma J, Shi C, Zhu H (2015). MiR-204-5p suppresses cell proliferation by inhibiting IGFBP5 in papillary thyroid carcinoma. Biochem Biophys Res Commun.

[41] Wang X, Li F, Zhou X (2016). MiR-204-5p regulates cell proliferation and metastasis through inhibiting CXCR4 expression in OSCC. Biomed Pharmacother.

[42] Gao W, Wu Y, He X, Zhang C, Zhu M, Chen B (2017). MicroRNA-204-5p inhibits invasion and metastasis of laryngeal squamous cell carcinoma by suppressing forkhead box C1. J Cancer.

[43] Yu CC, Chen PN, Peng CY, Yu CH, Chou MY (2016). Suppression of miR-204 enables oral squamous cell carcinomas to promote cancer stemness, EMT traits, and lymph node metastasis. Oncotarget.

[44] Wu Q, Zhao Y, Wang P (2018). MiR-204 inhibits angiogenesis and promotes sensitivity to cetuximab in head and neck squamous cell carcinoma cells by blocking JAK2-STAT3 signaling. Biomed Pharmacother.

[45] Iliopoulos D, Lindahl-Allen M, Polytarchou C, Hirsch HA, Tsichlis PN, Struhl K (2010). Loss of miR-200 inhibition of Suz12 leads to polycomb-mediated repression required for the formation and maintenance of cancer stem cells. Mol Cell.

[46] Zhang N, Zhang H, Liu Y, Su P, Zhang J, Wang X (2019). SREBP1, targeted by miR-18a-5p, modulates epithelial-mesenchymal transition in breast cancer via forming a co-repressor complex with Snail and HDAC1/2. Cell Death Differ.

[47] Phillips S, Kuperwasser C (2014). SLUG: critical regulator of epithelial cell identity in breast development and cancer. Cell Adh Migr.

[48] Cobaleda C, Perez-Caro M, Vicente-Duenas C, Sanchez-Garcia I (2007). Function of the zinc-finger transcription factor SNAI2 in cancer and development. Annu Rev Genet.

[49] Nieto MA (2002). The snail superfamily of zinc-finger transcription factors. Nat Rev Mol Cell Biol.

[50] Kasinath V, Faini M, Poepsel S, Reif D, Feng XA, Stjepanovic G (2018). Structures of human PRC2 with its cofactors AEBP2 and JARID2. Science.

[51] Hojfeldt JW, Laugesen A, Willumsen BM, Damhofer H, Hedehus L, Tvardovskiy A (2018). Accurate H3K27 methylation can be established de novo by SUZ12-directed PRC2. Nat Struct Mol Biol.

[52] Scully C, Field JK, Tanzawa H (2000). Genetic aberrations in oral or head and neck squamous cell carcinoma 2: chromosomal aberrations. Oral Oncol.

[53] Spafford MF, Koch WM, Reed AL, Califano JA, Xu LH, Eisenberger CF (2001). Detection of head and neck squamous cell carcinoma among exfoliated oral mucosal cells by microsatellite analysis. Clin Cancer Res.

[54] Bao W, Wang HH, Tian FJ, He XY, Qiu MT, Wang JY (2013). A TrkB-STAT3-miR-204-5p regulatory circuitry controls proliferation and invasion of endometrial carcinoma cells. Mol Cancer.

[55] Kim E, Kim M, Woo DH, Shin Y, Shin J, Chang N (2013). Phosphorylation of EZH2 activates STAT3 signaling via STAT3 methylation and promotes tumorigenicity of glioblastoma stem-like cells. Cancer cell.

[56] Yao C, Su L, Shan J, Zhu C, Liu L, Liu C (2016). IGF/STAT3/NANOG/Slug signaling axis simultaneously controls epithelial-mesenchymal transition and stemness maintenance in colorectal cancer. Stem cells.

[57] Fan TF, Deng WW, Bu LL, Wu TF, Zhang WF, Sun ZJ (2017). B7-H3 regulates migration and invasion in salivary gland adenoid cystic carcinoma via the JAK2/STAT3 signaling pathway. Am J Transl Res.

[58] Zheng B, Chen L, Pan CC, Wang JZ, Lu GR, Yang SX (2018). Targeted delivery of miRNA-204-5p by PEGylated polymer nanoparticles for colon cancer therapy. Nanomedicine (Lond).

[59] Xu L, Kittrell S, Yeudall WA, Yang H (2016). Folic acid-decorated polyamidoamine dendrimer mediates selective uptake and high expression of genes in head and neck cancer cells. Nanomedicine (Lond).

[60] Li J, Wang CY (2008). TBL1-TBLR1 and beta-catenin recruit each other to Wnt target-gene promoter for transcription activation and oncogenesis. Nat Cell Biol.

[61] Zhuang Z, Xie N, Hu J, Yu P, Wang C, Hu X (2017). Interplay between DeltaNp63 and miR-138-5p regulates growth, metastasis and stemness of oral squamous cell carcinoma. Oncotarget.

[62] Dallavalle C, Albino D, Civenni G, Merulla J, Ostano P, Mello-Grand M (2016). MicroRNA-424 impairs ubiquitination to activate STAT3 and promote prostate tumor progression. J Clin Invest.

[63] Morrissey ER, Juarez MA, Denby KJ, Burroughs NJ (2011). Inferring the time-invariant topology of a nonlinear sparse gene regulatory network using fully Bayesian spline autoregression. Biostatistics.

[64] Ben-Porath I, Thomson MW, Carey VJ, Ge R, Bell GW, Regev A (2008). An embryonic stem cell-like gene expression signature in poorly differentiated aggressive human tumors. Nat Genet.

[65] Mistry DS, Chen Y, Wang Y, Zhang K, Sen GL (2014). SNAI2 controls the undifferentiated state of human epidermal progenitor cells. Stem Cells.

[66] Li JH, Liu S, Zhou H, Qu LH, Yang JH (2014). StarBase v2.0: decoding miRNA-ceRNA, miRNA-ncRNA and protein-RNA interaction networks from large-scale CLIP-Seq data. Nucleic Acids Res.

