# Supplementary material for: MicroRNA-204-5p is a tumor suppressor and potential therapeutic target in head and neck squamous cell carcinoma
Source: Theranostics. 2020 Jan 1;10(3):1433–53. doi: 10.7150/thno.38507 (PMC6956807; doi:10.7150/thno.38507)
Supplement: Supplementary file 1 — Supplementary figures and tables. [file thnov10p1433s1.pdf]

Figure S1

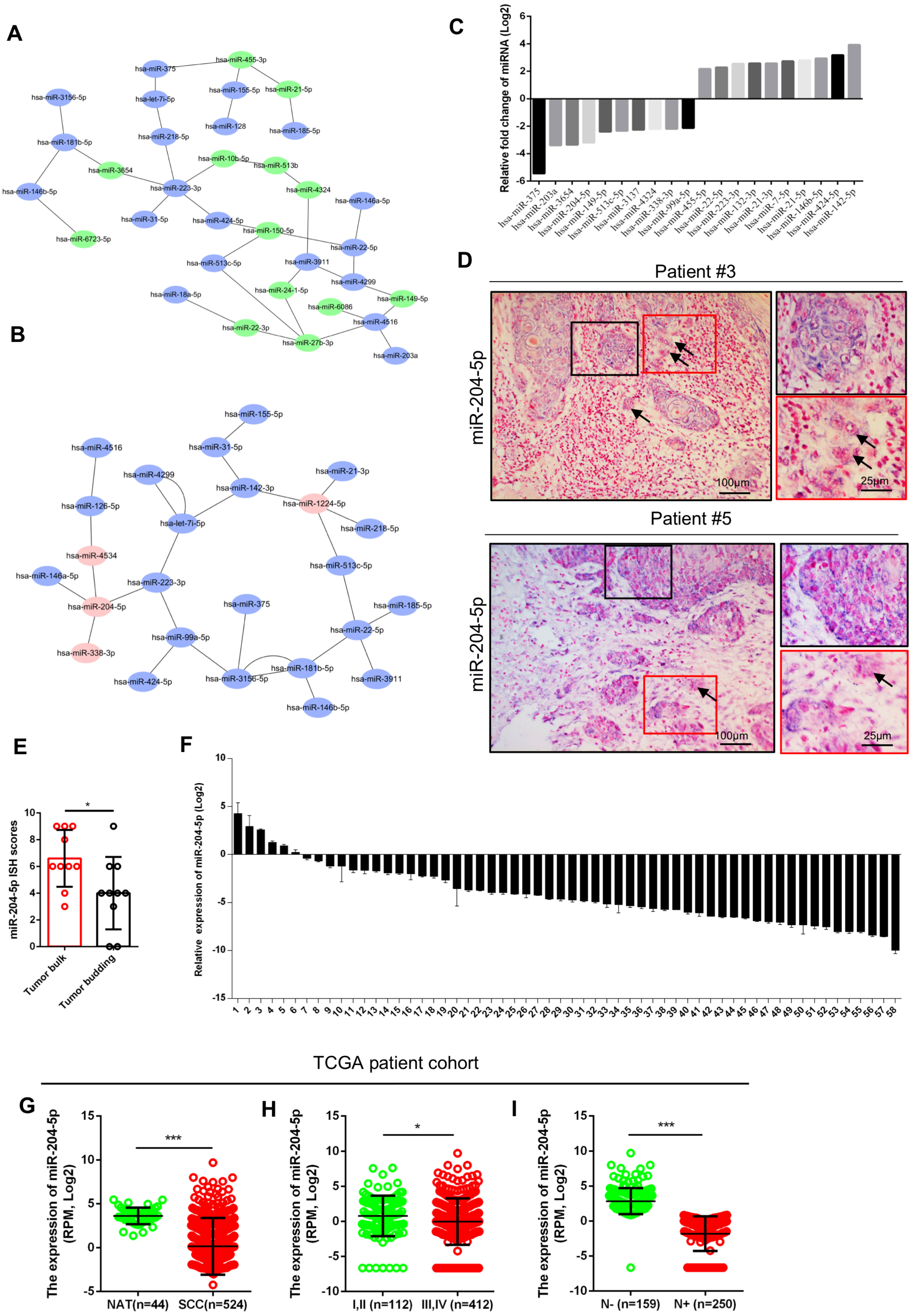
A
B
C
D
E
F
G
H
I
Relative fold change of miRNA (Log2)
Patient #3
Patient #5
miR-204-5p
100μm
25μm
miR-204-5p ISH scores
Tumor bulk
Tumor budding
Relative expression of miR-204-5p (Log2)
TCGA patient cohort
The expression of miR-204-5p (RPM, Log2)
NAT(n=44)
SCC(n=524)
I,II (n=112)
III,IV (n=412)
N- (n=159)
N+ (n=250)

Figure S1. MiR-204-5p is downregulated in HNSCC and correlative with malignant progression of HNSCC. (A) Bayes network analysis for miRNA in NAT. The green color represents a miRNA specific expressing in NAT, and the blue color represents the common miRNA in both NAT and HNSCC. (B) Bayes network analysis for miRNA in HNSCC. The red color represents a miRNA specific expressing in HNSCC, and the blue color represents the common miRNA in both NAT and HNSCC. (C) The top ten down-regulation and up-regulation miRNA in 10 HNSCC samples as compared to paired NAT. Bars represent the relative expression levels of each miRNA from array. (D) Representative image of miR-204-5p expression in tumor budding of HNSCC. Tumor budding was indicated with black arrows. (E) The expression of miR-204-5p was decreased in tumor budding as compared to tumor bulk. (F) The expression of miR-204-5p in 58 paired HNSCC samples comparing with NATs. (G) Analysis of miR-204-5p expression in 44 NATs and 524 HNSCC samples from TCGA database, and the HNSCC samples were grouped by the clinical stage (H) and lymph node metastasis (I). Data with pathological lymph node status are available in 409 patients. Data represent mean ± SD. *P < 0.05 and ***P < 0.001 by Student's t test.

Figure S2

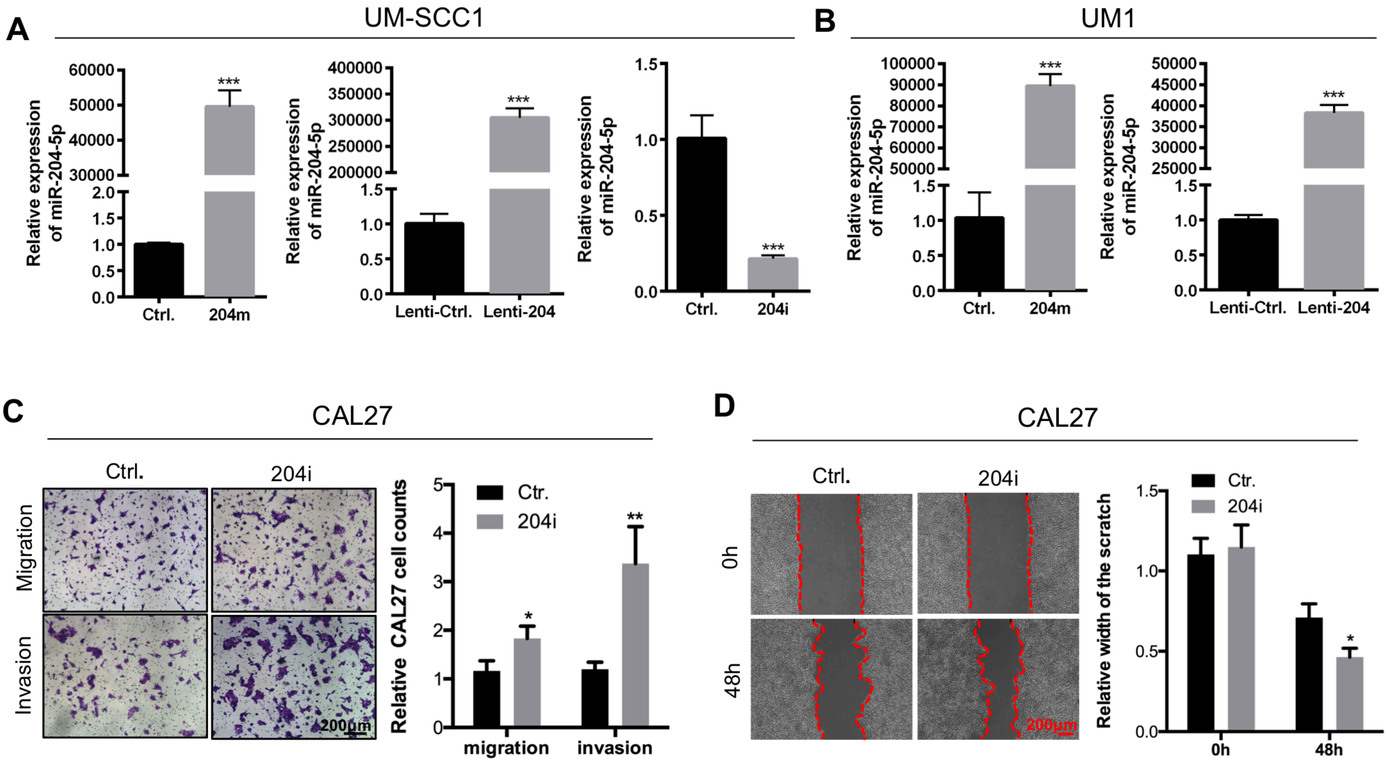

Figure S2. MiR-204-5p inhibits migration and invasion of HNSCC *in vitro*. (A, B) The miR-204-5p transfection efficiency was determined in HNSCC cells lines. (C) Cell migratory and invasive capacities was evaluated in HNSCC cells treated with miR-204-5p inhibitors and control inhibitors. (D) The ability of cell motility was evaluated by wound healing assay in HNSCC cells treated with miR-204 inhibitors and control inhibitors. Data represent mean ± SD. * $P < 0.05$, ** $P < 0.01$ by Student's t test.

Figure S3

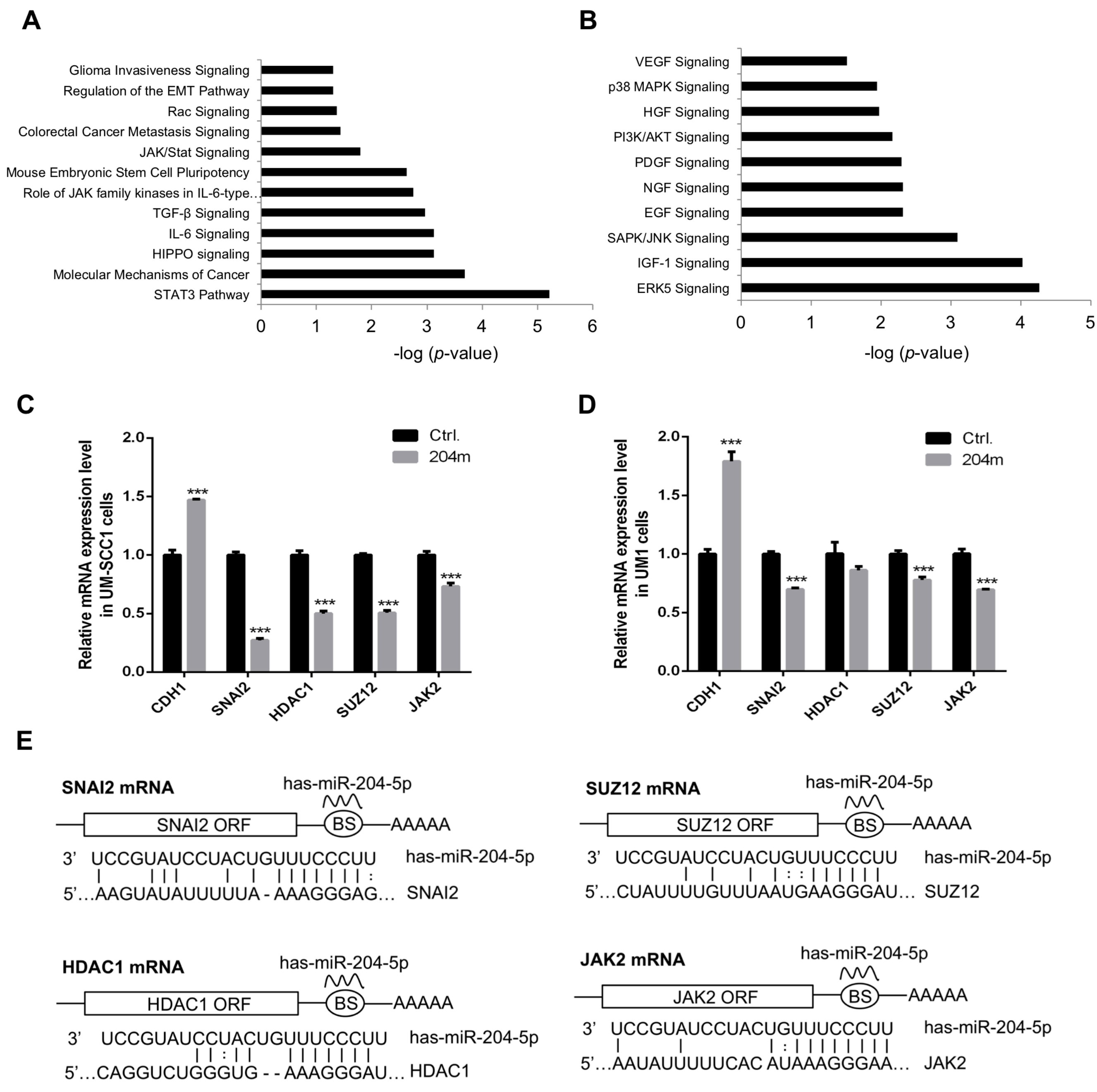

Figure S3. MiR-204-5p suppresses EMT and STAT3 pathway by targeting SNAI2, SUZ12, HDAC1 and JAK2. (A) IPA showing that miR-204-5p impacted several pathways involved in metastasis and stemness, including STAT3 and EMT pathway. (B) Major signaling affected by miR-204-5p expression in UM-SCC1 cells which were associated cell proliferation. (C, D) RT-qPCR analysis of CDH1, SNAI2, HDAC1, SUZ12 and JAK2 mRNA expression level in HNSCC cells treated with miR-204-5p mimics. (E) Predicted binding sites in 3'UTRs of SNAI2, SUZ12, HDAC1 and JAK2 mRNA. Data represent mean ± SD. ***P < 0.001 by Student's t test.

Figure S4

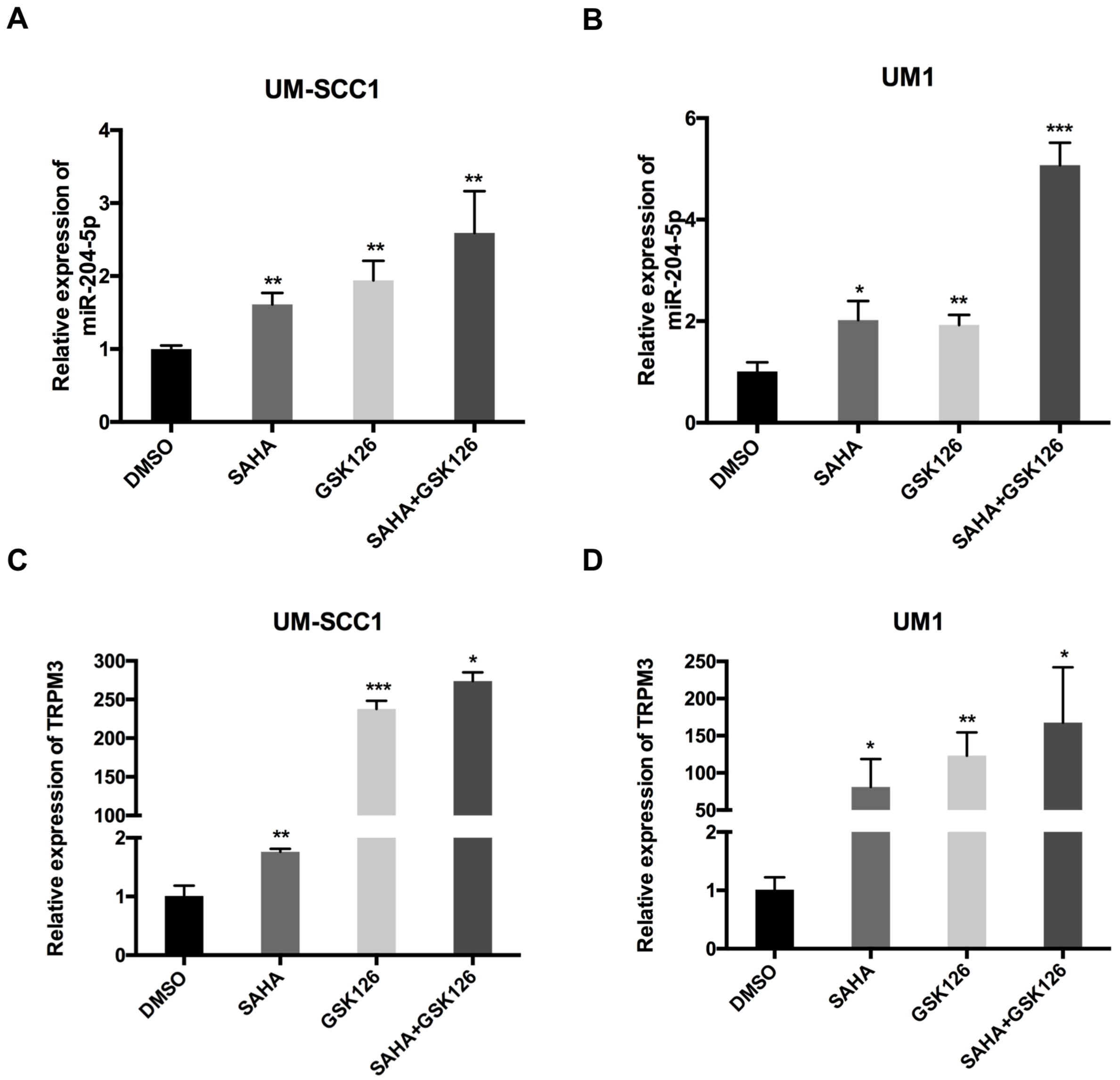

Figure S4. MiR-204-5p and its host gene are suppressed by SNAI2/PRC2/HDAC1 complex. (A, B) RT-qPCR analysis of miR-204-5p expression level in HNSCC cells at 24 h after treatment with HDAC1 inhibitor SAHA (3 μM), or EZH2 inhibitor GSK126 (5 μM), or their combination. (C, D) RT-qPCR analysis of TRPM3 expression level in HNSCC cells at 24 h after treatment with SAHA (3 μM), or GSK126 (5 μM), or their combination. Data represent mean ± SD. **P* < 0.05, ***P* < 0.01 and ****P* < 0.001 by Student's t test.

Figure S5

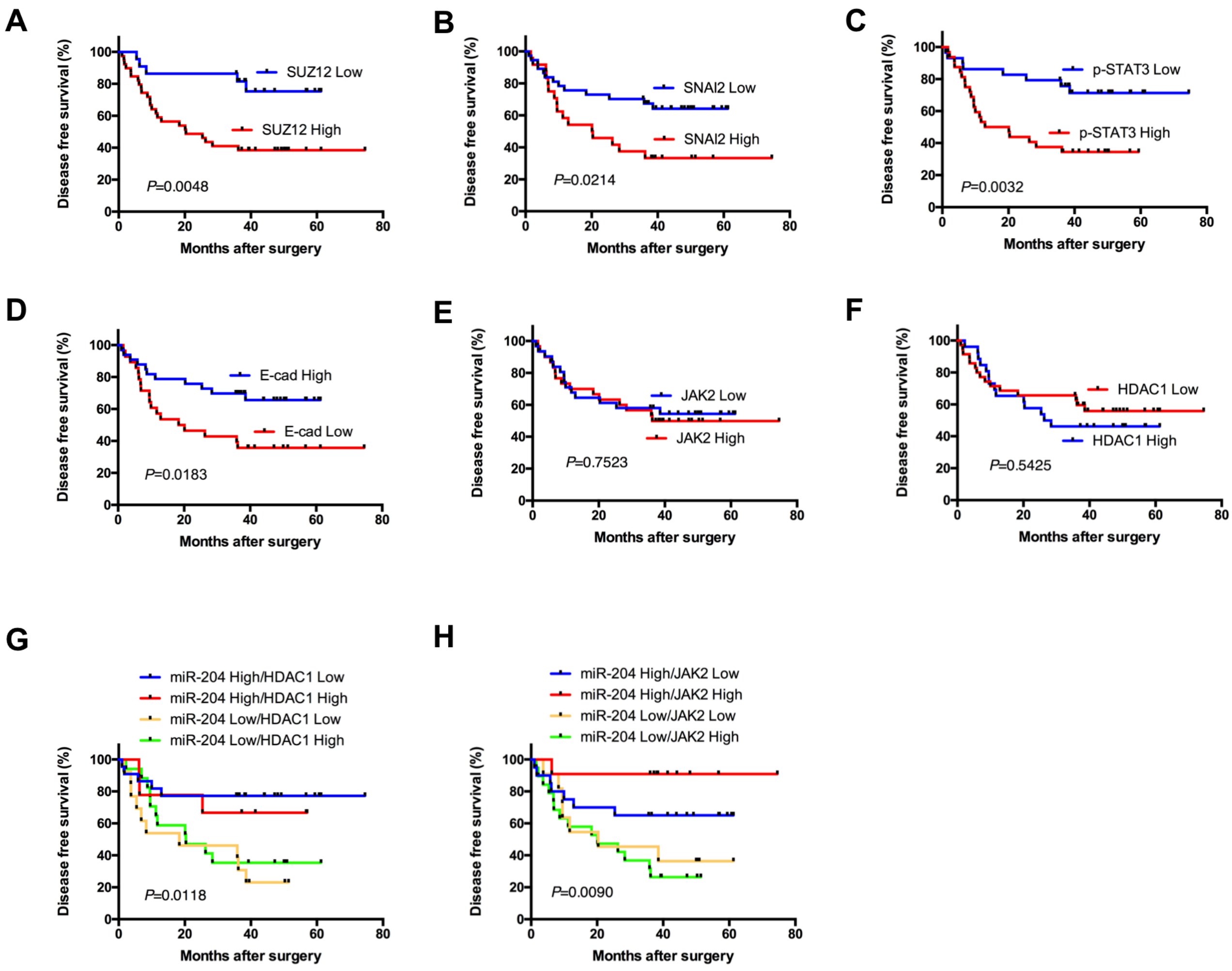

Figure S5. Decrease of miR-204-5p indicates a poor prognosis in HNSCC patients. Kaplan–Meier curves for survival of patients with HNSCC that grouped by the expression of miR-204-5p related genes, including SUZ12 (A), SNAI2 (B), p-STAT3 (C), E-cadherin (D), JAK2 (E), and HDAC1 (F). (G, H) Kaplan–Meier analyses for survival of patients with HNSCC that have expression levels of miR-204-5p high or low combined with HDAC1 and JAK2 high or low. *P* values of Kaplan–Meier analyses were calculated using log-rank and Gehan–Breslow–Wilcoxon tests.

Figure S6

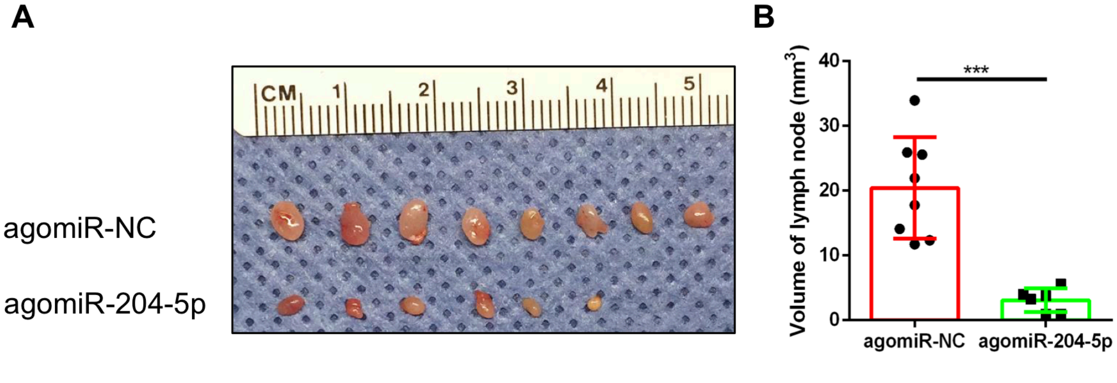

Figure S6. AgomiR-204-5p inhibits tumor progression in PDX model. Dissected cervical lymph node (A) and quantitative analysis of cervical lymph node volume (B) harvested from orthotopic PDX treated with agomiR-204-5p and agomiR-NC. Values are mean ± SD. n=5. ****P* < 0.001 by Student's t test.

Table S1: Characteristics of HNSCC cell lines.

Cell lines	Primary sites	Age at sampling	Gender
UM-SCC1	Floor of mouth	72Y	Male
UM1	Tongue	47Y	Male
SCC9	Tongue	25Y	Male
SCC15	Tongue	55Y	Male
SCC25	Tongue	70Y	Male
HSC3	Tongue	64Y	Male
HSC6	Oral cavity	NA	NA
CAL27	Tongue	56Y	Male
CAL33	Tongue	69Y	Male

NA: data not available.

Table S2: The clinicopathological features of HNSCC patients.

Patient ID	Diagnosis	Gender	Age (yr)	TNM stage
1	tongue squamous cell carcinoma	female	60	T4N0M0
2	tongue squamous cell carcinoma	male	33	T1N0M0
3	tongue squamous cell carcinoma	male	65	T2N2cM0
4	tongue squamous cell carcinoma	male	39	T3N1M0
5	tongue squamous cell carcinoma	female	27	T2N0M0
6	tongue squamous cell carcinoma	male	58	T1N0M0
7	squamous cell carcinoma of floor of mouth	male	47	T3N2M0
8	tongue squamous cell carcinoma	female	50	T2N0M0
9	squamous cell carcinoma of the gingiva	male	68	T3N2M0
10	squamous cell carcinoma of floor of mouth	male	59	T4N2M0
11	tongue squamous cell carcinoma	female	37	T3N0M0
12	tongue squamous cell carcinoma	male	47	T2N1M0
13	tongue squamous cell carcinoma	female	57	T3N1M0
14	tongue squamous cell carcinoma	female	58	T2N1M0
15	oropharyngeal squamous cell carcinoma	male	70	T3N1M0
16	tongue squamous cell carcinoma	female	50	T2N1M0
17	tongue squamous cell carcinoma	male	45	T2N0M0
18	tongue squamous cell carcinoma	female	50	T2N0M0
19	tongue squamous cell carcinoma	female	46	T3N0M0
20	tongue squamous cell carcinoma	male	49	T1N0M0
21	tongue squamous cell carcinoma	male	32	T2N0M0
22	tongue squamous cell carcinoma	female	62	T3N0M0
23	tongue squamous cell carcinoma	female	49	T2N1M0
24	tongue squamous cell carcinoma	male	73	T2N1M0
25	squamous cell carcinoma of the gingiva	female	70	T3N0M0
26	tongue squamous cell carcinoma	male	70	T3N2M0
27	squamous cell carcinoma of floor of mouth	male	50	T3N1M0
28	tongue squamous cell carcinoma	male	57	T2N0M0
29	tongue squamous cell carcinoma	female	60	T1N0M0

30	tongue squamous cell carcinoma	male	49	T3N0M0
31	tongue squamous cell carcinoma	female	65	T1N0M0
32	tongue squamous cell carcinoma	female	30	T2N0M0
33	oropharyngeal squamous cell carcinoma	male	55	T2N0M0
34	squamous cell carcinoma of floor of mouth	male	57	T3N1M0
35	tongue squamous cell carcinoma	male	60	T1N0M0
36	buccal squamous cell carcinoma	male	49	T4N2M0
37	squamous cell carcinoma of floor of mouth	male	64	T4aN1M0
38	tongue squamous cell carcinoma	male	33	T2N1M0
39	tongue squamous cell carcinoma	female	47	T3N1M0
40	tongue squamous cell carcinoma	female	41	T2N1M0
41	tongue squamous cell carcinoma	female	54	T2N0M0
42	tongue squamous cell carcinoma	male	47	T2N0M0
43	tongue squamous cell carcinoma	male	64	T3N0M0
44	tongue squamous cell carcinoma	male	34	T2N2bM0
45	tongue squamous cell carcinoma	male	43	T4N0M0
46	tongue squamous cell carcinoma	female	32	T2N2cM0
47	tongue squamous cell carcinoma	male	59	T4aN2aM0
48	tongue squamous cell carcinoma	male	52	T2N1M0
49	tongue squamous cell carcinoma	male	40	T2N2bM0
50	tongue squamous cell carcinoma	female	64	T2N0M0
51	tongue squamous cell carcinoma	female	32	T2N0M0
52	tongue squamous cell carcinoma	male	39	T2N0M0
53	squamous cell carcinoma of floor of mouth	male	49	T4N0M0
54	tongue squamous cell carcinoma	male	46	T1N0M0
55	tongue squamous cell carcinoma	female	63	T2N0M0
56	tongue squamous cell carcinoma	female	44	T3N0M0
57	squamous cell carcinoma of the gingiva	female	77	T4N0M0
58	tongue squamous cell carcinoma	female	42	T1N0M0
59	squamous cell carcinoma of the gingiva	female	55	T2N0M0
60	squamous cell carcinoma of floor of mouth	male	65	T2N0M0
61	squamous cell carcinoma of the gingiva	male	65	T2N0M0

Table S3: List of primers.

Genes	Sequences	Assay
SNAI2	Forward: ATCTGCGGCAAGGCGTTTTCCA<br>Reverse: GAGCCCTCAGATTTGACCTGTC	qPCR
HDAC1	Forward: AATGGAGATGTTCCAGCCTAGTGCG<br>Reverse: CACACTTGGCGTGTCCTTTGATA	qPCR
SUZ12	Forward: GCCCTGTGTTATATGTGTTTCACG<br>Reverse: GGTGCTTGTTAATGTGCCAGTAG	qPCR
JAK2	Forward: CCAGATGGAAACTGTTCGCTCAG<br>Reverse: GAGGTTGGTACATCAGAAACACC	qPCR
CDH1	Forward: GCCTCCTGAAAAGAGAGTGGAAG<br>Reverse: TGGCAGTGTCTCTCCAAATCCG	qPCR
TRPM3	Forward: CCAAGCTCAGCATCTGTTCA<br>Reverse: TCCTGGTGTCCTTCTGATCC	qPCR
CDH1-EBOX	Forward: ACTCCAGGCTAGAGGGTCAC<br>Reverse: GCCCGACCCGACCGCACCCG	ChIP-PCR
CDH1-NEG	Forward: GCTGTGCCCAGCCTCCATGTTTTA<br>Reverse: CAGGAGATGAAAGAGGGAAGCCA	ChIP-PCR
MIR-204-P1	Forward: TACCATATAGGACAGCTTGACGG<br>Reverse: GAAAATGCCTAGCATGGTGCTTG	ChIP-PCR
TRMP3-TSS	Forward: ACAGGCTGTTTTGTGCAGGCTGTC<br>Reverse: ATGTGGCTGAATGGAGGCACACT	ChIP-PCR
MIR-204-TSS	Forward: TTCCTGATCGCGTACCCATGGCTA<br>Reverse: TGCCTTCCCAGCCTCCTTCATAT	ChIP-PCR
TRMP3-NEG	Forward: GGTGAGTGATGTTTAATAGCCAGG<br>Reverse: CCAACTCCATGTTGTGGCATCAT	ChIP-PCR
SNAI2	Forward: ACTCCATTACATGTCGGTTGTCTG<br>Reverse: CATTCTCCTGTGTTTTGTTCTTG	RIP-PCR
HDAC1	Forward: GACAAACTCCTGAAATGCCAAGTG<br>Reverse: ACAGGAGATCCTTGAAGACCCAG	RIP-PCR
SUZ12	Forward: TTGGAAACAGATAGTGTCTCAGGG<br>Reverse: CATCTAACAGTGCCTGTTTGGAAC	RIP-PCR
JAK2	Forward: GCCAGCATTATAAGCAGGTGTAT<br>Reverse: TGTATTATTTAGGGTCAGGGAAA	RIP-PCR

Table S4: List of primary antibodies.

Antibodies	Source	Identifier
SUZ12 (Polyclonal)	Abcam	Cat#ab12073
SNAI2 (Clone C19G7)	Cell Signaling Tech	Cat#9585
E-Cadherin (Clone 24E10)	Cell Signaling Tech	Cat# 3195
HDAC1 (Clone D5C6U)	Cell Signaling Tech	Cat# 34589
STAT3 (Clone D3Z2G)	Cell Signaling Tech	Cat#12640
Phospho-STAT3 (Tyr705) (Clone D3A7)	Cell Signaling Tech	Cat#9145
JAK2 (Clone D2E12)	Cell Signaling Tech	Cat#3230
EZH2 (Clone D2C9)	Cell Signaling Tech	Cat #5246
H3K27me3 (Clone mAbcam 6002)	Abcam	Cat#ab6002
FLAG (Clone M2)	Sigma-Aldrich	Cat#F1804
HA (Clone 16B12)	Covance	Cat# MMS-101P
α-Tubulin (Clone 11H10)	Cell Signaling Tech	Cat#2125
Pan-Cytokeratin	Novus	Cat#NBP1-22909
AGO2	Abcam	Cat#ab32381
β -actin	Abcam	Cat#ab8227
STAT3 (Clone 124H6)	Cell Signaling Tech	Cat# 9139
p-STAT3 (Clone D3A7)	Cell Signaling Tech	Cat# 9145
